# Proteomic analysis of laser capture microscopy purified myotendinous junction regions from muscle sections

**DOI:** 10.1186/1477-5956-12-25

**Published:** 2014-05-07

**Authors:** Tugba Can, Laura Faas, David A Ashford, Adam Dowle, Jerry Thomas, Peter O’Toole, Gonzalo Blanco

**Affiliations:** 1 Department of Biology, University of York, Wentworth Way, York YO10 5DD, UK; 2 Bioscience Technology Facility, Department of Biology, University of York, Wentworth Way, York YO10 5DD, UK

## Abstract

The myotendinous junction is a specialized structure of the muscle fibre enriched in mechanosensing complexes, including costameric proteins and core elements of the z-disc. Here, laser capture microdissection was applied to purify membrane regions from the myotendinous junctions of mouse skeletal muscles, which were then processed for proteomic analysis. Sarcolemma sections from the longitudinal axis of the muscle fibre were used as control for the specificity of the junctional preparation. Gene ontology term analysis of the combined lists indicated a statistically significant enrichment in membrane-associated proteins. The myotendinous junction preparation contained previously uncharacterized proteins, a number of z-disc costameric ligands (e.g., actinins, capZ, αB cristallin, filamin C, cypher, calsarcin, desmin, FHL1, telethonin, nebulin, titin and an enigma-like protein) and other proposed players of sarcomeric stretch sensing and signalling, such as myotilin and the three myomesin homologs. A subset were confirmed by immunofluorescence analysis as enriched at the myotendinous junction, suggesting that laser capture microdissection from muscle sections is a valid approach to identify novel myotendinous junction players potentially involved in mechanotransduction pathways.

## Background

Laser-assisted cell microdissection in combination with laser-pressure catapulting (commonly referred to as laser capture microdissection or LCM) has been exploited for over a decade to isolate pure population of cells, specific regions from tissue sections or even single chromosomes [1-4]. LCM has also been applied to identify the unique expression profiles of specialized regions within complex cells. For example, LCM has been combined with RNA isolation and transcriptome analysis to identify specific transcripts and components of the neuromuscular junction [5,6]. In combination with liquid chromatography tandem mass spectrometry (LC-MS/MS), LCM has been extensively used, amongst others applications, to purify and profile cancer cells (for a review see [7]), profile plaques in neurological disease [8-11] or elucidate the expression profile of inclusion bodies within muscle fibres [12]. In the experimental process of an expression profiling, LCM is often the limiting step, given the length of time required to recover a small amount of material. However, when combined with high-resolution mass spectrometers, LCM of tissue sections can potentially identify hundreds of proteins from as little as 1000 cells [13].

In an attempt to identify candidate proteins for mechanotransduction processes, we decided to focus on the myotendinous junction (MTJ). Like the neuromuscular junction, the myotendinous junction is also a highly specialized anatomical region of the muscle fibre thought to be controlled by specialized underlying nuclei. The MTJ encompasses an alignment of protein complexes from the subsarcolemmal cytoskeleton, through the sarcolemma and basal lamina, to the collagen matrix on the tendinous side of the junction (see [14] for a recent review on the development and organization of the MTJ). Actin filaments bundled with alpha-actinin and desmin project from the electrodense terminal sarcomeric z-discs [15] towards the sarcolemma. At the sarcolemma, they interact with the dystrophin-associated and the α7β1 integrin protein complexes, which in turn connect with the extracellular matrix through the basal lamina protein laminin, following a similar arrangement to the costamere [16-20]. The MTJ is thus a major site of force transmission from myofibrils to the extracellular matrix and adapts to mechanical stress by increasing the muscle-tendon contact area [21]. Indeed, the myotendinous junction is enriched in costameric proteins and core elements of the z-disc.

In recent years, the sarcomeric z-disc has emerged as a plausible structure that mediates adaptive responses to mechanical stresses. Such notion has evolved from the discovery of proteins over the last two decades that, when mutated or lost from the z-disc, provoke skeletal muscle and heart disorders. Characterizations of the muscle phenotypes in these disorders have demonstrated that the z-disc is not just a structural link between sarcomeres, but that it contains both a structural scaffold for modulating biological sensors [22] and signalling hubs for mechanosensation and mechanotransduction [23,24]. Moreover, the presence within the z-disc of the kyphoscoliosis peptidase protein KY, a protein required for the muscle hypertrophic response to chronic overload [25,26], suggests that the z-disc could also contain mechanistic triggers of hypertrophy.

Although the z-disc would be an ideal structure to profile in order to identify new players of mechanoreception and transduction, purification of z-discs that retain their intact physiological interactions is a challenging task. Biochemical enrichments of the z-disc have been successfully done using strong treatments (e.g., [27]). While these preparations have been useful to elucidate the cytoarchitecture of the purified insoluble material, most of the soluble associated proteins are lost during the extraction process, therefore defeating the purpose of a follow up proteomic analysis. On the other hand, purification of the intact z-disc by laser capture microdissection using thick longitudinal sections of muscle fibres is not possible because the width of the z-band is only 30–100 nm in the vertebrate muscle fibre [28], which is far beyond the resolution of LCM technology. As a potential alternative, we evaluate here the ability of LCM combined with LC-MS/MS analysis to profile the myotendinous junction region.

## Results

### LCM based purification of membrane sections

**Figure 1:**
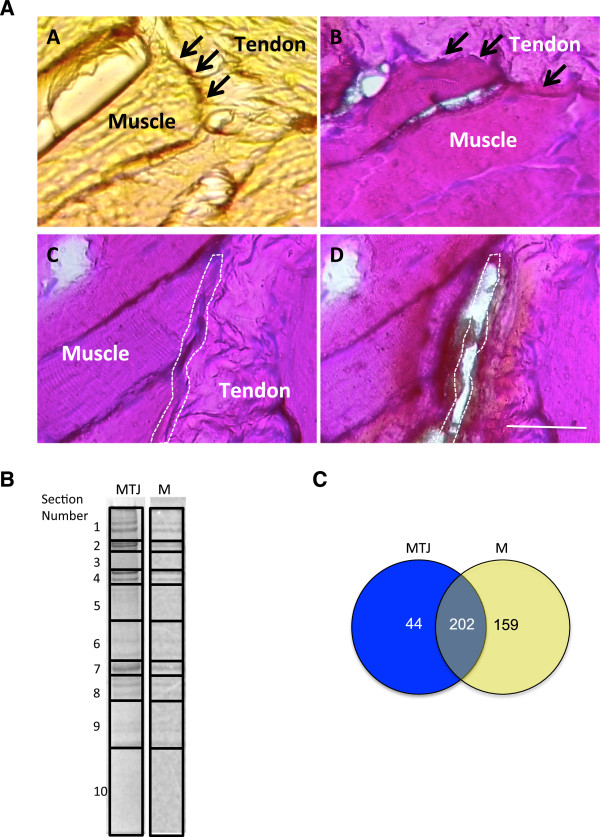
**Proteomic analysis of laser capture microdissection of myotendinous junction regions. A)** A comparative view of the myotendinous junction of a non-stained section **(panel A)** and an H&E stained section **(panel B)**, with tendon and muscle regions identified as indicated and myotendinous regions identified by arrows. Panels C and D show a representative example of a myotendinous junction selected region (white dotted line) selected for microdissection before **(panel C)** and after **(panel D)** laser application and catapulting. All pictures taken with a 40X objective. Scale bar indicates 50 microns. **B)** Protein gel illustrating sections excised for LC-MS/MS analysis. The grid overlaying the lanes of solubilized material from myotendinous region (MTJ) or extrajunctional membrane (M) indicates the gel blocks used for in-gel tryptic digestions. **C)** A Venn diagram indicating the number of proteins identified according to their presence in the myotendinous junction (MTJ) or the extrajunctional membrane region (M).

Mouse muscles that were processed included gastrocnemius, soleus, extensor digitorium longus, tibialis anterior and biceps. Fresh frozen tissue sections were used, instead of formalin-fixed and paraffin-embedded samples, to avoid any confounding factor that might be introduced by unequal fixation of the tissues. Mounting of the frozen muscles and processing by laser capture microdissection was carried as described in Methods. To facilitate the identification of the transition from the myofibre to the collagen matrix, a mild hematoxylin and eosin staining (H&E) was applied on sections spanning the myotendinous junctions (Figure 1A). This step also rendered the sections slightly drier and facilitated UV absorption, in our hands resulting in much more efficient catapulting of the tissue cuts from the slide into the collection tube. Given the narrow sarcolemmal regions selected for LCM purification (see a representative cut in Figure 1A, panels C and D), collecting enough protein for successful proteomic analysis would have been impractical using the recommended section thickness (8–12 μm). Therefore, sections ranging from 12 μm to 30 μm were tested for their ability to be catapulted into the collection tubes following laser dissection. Under the fixation and staining protocol applied, sections of up to 20 μm could be efficiently catapulted. Energy levels and focus of the laser were also tested and adjusted to the specific regions (see Methods for details). In total, 800 cuts were collected from the myotendinous junction and a similar amount from the extra junctional membrane regions, hereafter referred to as samples MTJ and M, respectively.

### Proteomic processing

The sectioned areas were as restricted as possible to the membrane (Figure 1A), therefore yielding very limited material that could not be subjected to further biochemical fractionations. Instead, all of the collected materials from samples MTJ and M were resuspended in SDS sample buffer and run into a polyacrylamide gel, which was subsequently Coomassie stained and cut into sections as illustrated in Figure 1B. Protein identification was performed by LC-MS/MS (see Methods for details). A total of 405 proteins (excluding keratins) were identified which included unique and common hits to samples MTJ and M. These identifications were classified as 44 IDs unique to the myotendinous junction, 159 IDs unique to the peripheral membrane and 202 IDs common to both preparations (Figure 1C). Table 1 shows a selection of proteins identified in the MTJ sample. The full lists are presented in Additional file 1: Table S1.

**Table 1:** Selection of proteins identified in the MTJ sample

**Connective tissue**			**Transmembrane**		
**Protein**	**Accession**	**Mascot score**	**Protein**	**Accession**	**Mascot score**
Biglycan	IPI00123194	396	Vdaxc1	IPI00230540	1135
Fibromodulin	IPI00120187	1487	Vdac2	IPI00122547	116
Mimecan	IPI00120848	96	Vdac3	IPI00122548	413
Col1a2	IPI00222188	3023	Mpz	IPI01008333	102
Col6a3	IPI00830749	1012			
Col6a2	IPI00621027	192			
Col1a1	IPI00329872	1357			
Col6a1	IPI00339885	861			
Col12a1	IPI00121430	767			
Prolargin	IPI00122293	718			
Laminin	IPI00756745	33			
**Intracellular membrane associated**			**Z-disc/costameric ligands**		
**Protein**	**Accession**	**Mascot score**	**Protein**	**Accession**	**Mascot score**
					
Vinculin	IPI00405227	18	Actinin	IPI00136701	4955
Plectin	IPI00229509	249	CapZ	IPI00111265	192
Desmin	IPI00130102	1462	α-cristallin	IPI00138274	93
Plakoglobin	IPI00229475	273	FilaminC	IPI00664670	138
Desmoplakin	IPI00553419	16	Cipher	IPI00403040	547
Asap2	IPI00355808	16	FHL1	IPI00309997	18
			Telethonin	IPI00119331	24
			Nebulin	IPI00720238	76
			Titin	IPI00986455	19546
			Enigma-like	IPI00415684	108
			Myotilin	IPI00120508	50
			Telethonin	IPI00119331	24
			Myoxenin-2	IPI00122334	18
			α-actin	IPI00114593	19409

As expected, the list of proteins common to MTJ and M contained extracellular matrix constituents (collagens I and VI), proteins involved in extracellular matrix assembly (biglycan, fibromodulin, mimecan), anchoring of the basement membrane (prolargin, laminin), transmembrane anion channels (Vdac 1, 2 and 3) and cytoskeletal anchoring proteins (e.g., vimentin). Achievement of specificity was suggested by the fact that most hits in the full lists were proteins with a function in muscle. To further test whether LCM purification resulted in the intended enrichment of intracellular and extracellular membrane-associated proteins, we searched for evidence of statistically significant enrichment of GO terms in the MTJ list. To avoid biasing the analysis towards membrane associated extracellular components, all keratins were eliminated from the list, as they were presumed to be contaminants. The 246 hits were mapped to 215 *Mus musculus* supervised entries in UniProtKB (http://www.uniprot.org/). This list was then submitted to the software package *GOrilla*[29], which confirmed that 211 out of the 215 genes were associated with GO terms. Finally, computerized analysis of the GO terms, using as reference the reviewed Mus musculus uniprot protein list (16665 proteins), showed that several categories corresponding to extracellular, membrane bound, z-disc and mitochondrial associated were indeed enriched at *p* values ranging between 10^−5^ and 10^−43^ (Additional file 2: Figure S1; full lists of genes per enriched GO term with the corrected *p* values for multiple testing [30] are shown in Additional file 3: Table S2). Analysis of the list specific to the M sample produced identical categories of GO term enrichment (data not shown).

### Identification of myotendinous junction proteins

To assess the quality of the myotendinous membrane preparation we looked for the presence of costameric proteins [20]. As summarized in Table 1, many costameric proteins were indeed present, including members of the connective tissue (collagens, laminin, prolargin, fibromodulin), intracellular (vinculin, g-actin, plectin, desmin) and a number of z-disc costameric ligands (actinins, CapZ, αΒ crystallin, filamin C, cypher, calsarcin, FHL1, telethonin, nebulin, titin and an enigma-like protein). Thus, the preparation from the junctional region contained a good representation of proteins integral to the costameric network. In addition, the myotendinous junction preparation contained unique hits to: components of desmosomes (plakoglobin), extracellular proteins (Alpha-2-HS-glycoprotein, Lyz1 Lysozyme C-1), proteins associated to the cytosolic face of the plasma membrane (the two annexin proteins Anxa1 and Anxa6) and an integral membrane protein (myelin). Many proteins were identified only in the M preparation (e.g., α-syntrophin, α-sarcoglycan) but, intriguingly, other transmembrane proteins of the dystrophin associated protein complex were absent from both preparations, perhaps reflecting a limitation of the non-fractionated approach undertaken (see also Discussion).

### Expression patterns of MTJ candidates

**Figure 2:**
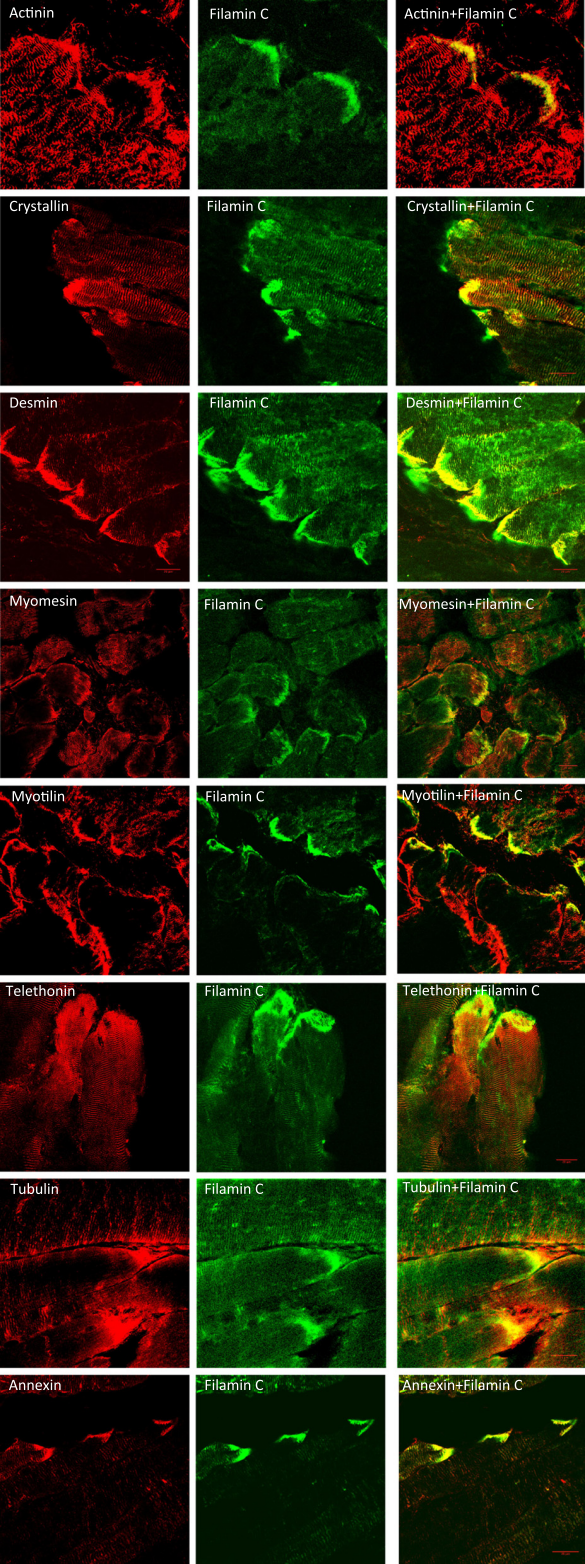
**Immunostainings of selected proteins from the MTJ sample.** Longitudinal sections of tibialis anterior muscle from 45 days old C57/Bl6 male were mice stained with antibodies against the indicated proteins. Strong staining of filamin C identifies the myotendinous junction region in each case. All pictures taken with a 63x oil lense. The scale bar indicates 20 microns.

**Figure 3:**
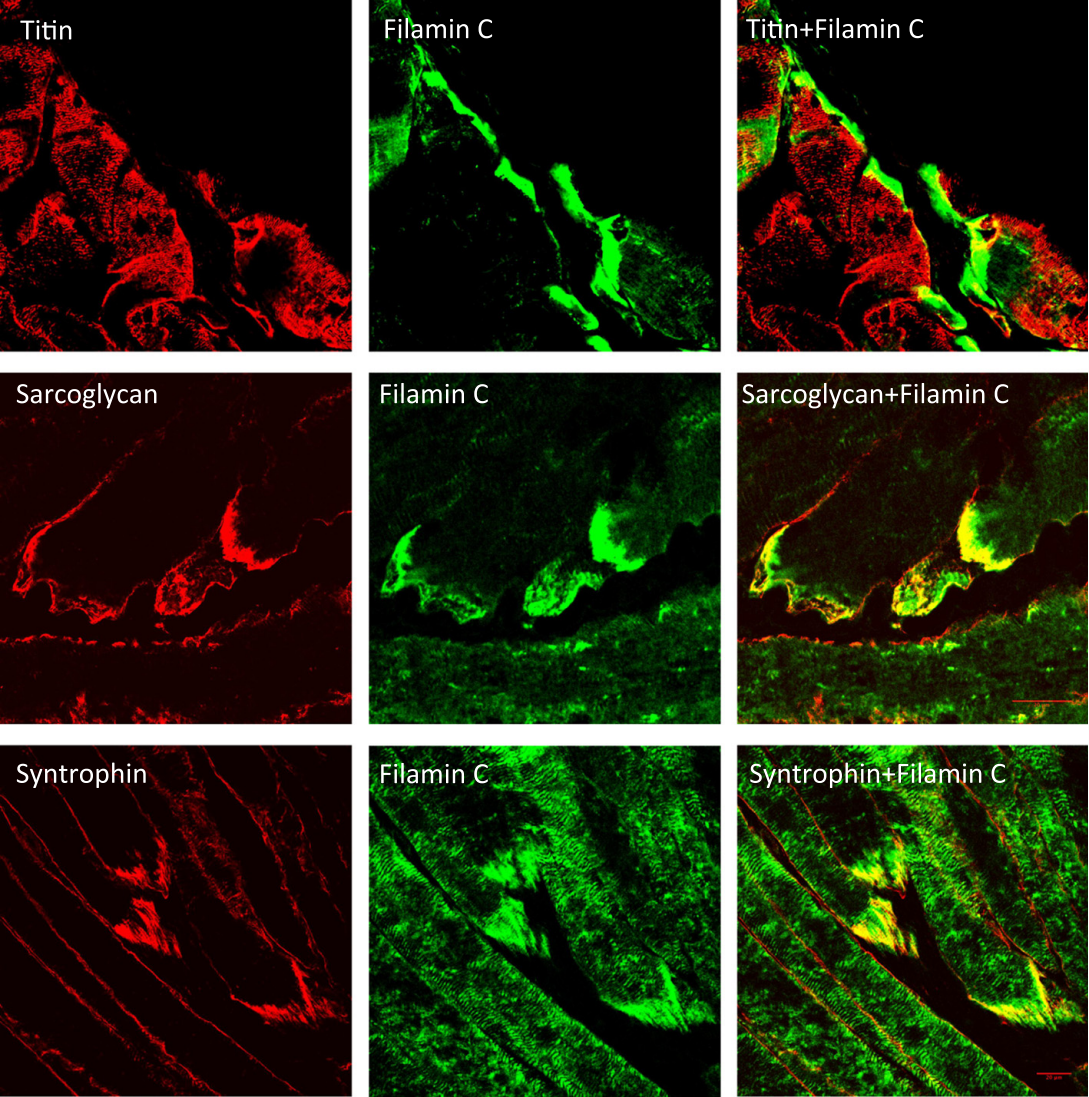
**Co-localization analysis of three proteins that were identified in the membrane sample only.** Titin shows very little co-localization with the myotendinous junction marker filamin C. The membrane-associated proteins α-sarcoglycan and α-syntrophin show partial co-localization. All pictures taken with a 63x oil lense. The scale bar indicates 20 microns.

Many of the proteins identified have been shown to have expression at sarcomeric or costameric level, but no information was available regarding their expression at the myotendinous junction. To confirm the quality of the results above, we tested the expression of some of the proteins identified by immunofluorescence for which commercial or published antibodies exist. Localization at the myotendinous junction was tested by co-localization with the well-established marker filamin C [31]. The results shown in Figure 2 indicates that for α-actinin, αΒ crystallin, desmin, myomesin, myotilin, telethonin, tubulin and annexin I (see Methods for antibody details), the expression was consistent with the proteomic results, since a good degree of co-localization with filamin C was obtained for all of them. Localization at the MTJ was anticipated for desmin [32] and α-actinin [33]. However, a stronger signal for α-crystallin, myomesin, myotilin, telethonin and annexin I at the myotendinous junction region had not been, to our knowledge, previously reported. Additionally, we tested three proteins that were identified in the M sample only: the giant sarcomeric protein titin 1 and the dystrophin associated protein complex members α-sarcoglycan (transmembrane) and α-syntrophin (cytoplasmic) (Figure 3). Titin 1 appeared to show very little co-localization with filamin C. In contrast, α-syntrophin and α-sarcoglycan gave stronger signal at the myotendinous junction compared to the extra junctional membrane. This was expected given that syntrophins and sarcoglycans are members of the dystrophin associated protein complex and dystrophin has been previously found at subsarcolemmal deposits at the junctional folds of the myotendon [34,35].

**Figure 4:**
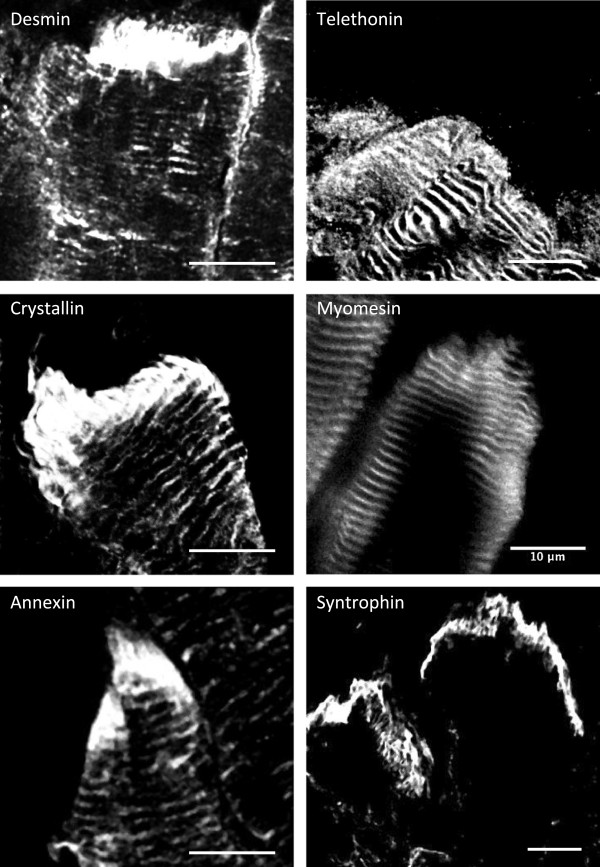
**Changes in subcellular localization patterns from the myofibre to the MTJ region illustrated for selected proteins.** Note that desmin, αB crystallin and anexin I appear to fill the NMJ region on the muscle side beyond their z-disc localization. Telethonin (z-disc) and myomesin (M line) appear to increase their concentration at the most terminal sarcomeres without changing their characteristic banding patterns. For comparison, the MTJ pattern of α-syntrophin is also shown. All pictures taken with a 63x oil lense with variables zoom factors applied. All scale bars indicate 10 microns.

Myomesin 1, 2 and 3 are major components of the M-band [36,37], therefore the presence of the three of them in the MTJ sample was unexpected. To distinguish between accumulations at the MTJ junctional folds from increased expression of the candidate protein at the terminal sarcomeres, higher resolution confocal views were obtained. The results shown in Figure 4 indicate that desmin, αΒ crystallin, annexin I and α-syntrophin appear to modify their expression pattern at this level and strongly stain the MTJ region. In contrast, as determined by co-stainings with α-actinin (data not shown), telethonin and myomesin remained restricted to the z-disc and the M-line, respectively (Figure 4). Thus, the presence of telethonin and myomesin in the MTJ sample may originate from their increased expression at the most terminal sarcomeres. Incubations of the sections with the secondary antibodies alone did not produce any detectably signal using identical incubation and confocal setting conditions.

## Discussion

The skeletal muscle mechanoreceptors and how specific mechanical inputs are converted into biochemical signals that trigger specific muscle adaptations remain elusive. In recent years, the sarcomeric z-disc has emerged as a plausible structure that mediates adaptive responses to mechanical stresses (e.g.,: [38,39]). This study aimed at testing the suitability of LCM from muscle sections in combination with LC/MS-MS for the identification of proteins associated with the myotendinous junction, since this structure contains z-disc material and is physically more approachable than the z-disc. Although there are obvious limitations to this approach, e.g., restriction of the analysis to soluble proteins or the physical resolution of the cuts, the proteomic analysis of the myotendinous junction and extra junctional membrane content showed a significant enrichment of GO-terms for intracellular and extracellular membrane-associated proteins. Indeed, despite H&E staining having been suggested to interfere with direct MALDI MS analysis of LCM captured cells [40], the protocol as described here was successful in identifying some differences between both membrane regions. Moreover, many costameric/z-disc proteins were identified in the myotendinous junction sample. The most likely reason why specific proteins previously found associated with the myotendinous junction were not identified here is that they were not present in sufficient abundance in the solubilized sample. For example, collagen VI, a protein enriched at the muscle endomysium [41], was successfully identified, but the less abundant collagen V [42] failed to be detected. Limited solubility or the fact that trypsin digestion may have also resulted in peptides outside the optimal size range or that do not ionise well and therefore are not suitable for MS, might explain why additional members of the costamere junction or other known MTJ proteins failed to be detected. It is also anticipated, given the size of the myotendinous junctional folds relative to the purified LCM cuts, that many of the identified proteins will not show a specific subcellular myotendinous junction localization.

A mechanotransduction mechanism for maintaining homeostasis in mechanically stressed cells has recently been proposed involving tension-induced targeted degradation of the actin crosslinker protein filamin and its upregulation [43,44]. Filamin C is highly enriched at the myotendinous junction and its ability to crosslink makes it a suitable candidate to sense mechanical stress, as tension can induce the exposition of cryptic interaction sites [45] and changes in the rates of protein turnover [43]. Since the immunoglobulin- and fibronectin-like repeats present in filamin are also found in many other muscle proteins with filament and myofibril crosslinking roles [46,47], adaptations to specific mechanical strains could also be mediated by other globular repeat containing proteins. In addition to filamin C, other proteins identified in the MTJ sample containing a succession of repeated domains included titin, nebulin, myosin binding protein C, obscurin, myotilin and the three myomesin isoforms. The presence of the three known M-band myomesin isoforms was intriguing. Myomesins share repeats of globular domains in their composition, have been proposed to participate in a stress-sensing mechanism and provide elasticity to the M-band [36,48,49]. Although myomesin accumulation at the myotendinous junction appears to be the case on the basis of the strong immunofluorescence signal detected at the edge of the muscle fibre, the signal remained restricted to the M-band at the terminal ends of the myofibrils (Figure 4). It would therefore be unlikely for myomesin to participate in membrane bound stretch complexes at the myotendinous junction, but it is plausible that their increased expression at this region reflects higher mechanical stress of the terminal sarcomeres. The same might be concluded for telethonin, as the stronger signal observed at the end of the fibre was not caused by accumulation beyond its characteristic z-disc expression. We therefore conclude that telethonin and myomesin do not have myotendinous junction specific functions, but that their presence in the proteomic MTJ sample is probably due to their increased expression at the most terminal sarcomeres.

Three proteins of diverse functions that have been previously associated with the z-disc were shown here to intensely stain the MTJ: myotilin, annexin I and αB crystallin. Myotilin is a z-disc protein that binds F-actin directly and bundles actin filaments in vitro [50]. However, despite the fact that mutations in the myotilin gene have been implicated in limb girdle muscular dystrophy 1A (LGMD1A) [51], myofibrillar myopathy (MFM) [52], and in a rare condition called spheroid body myopathy (SBM) [53], the function of myotilin in normal muscle physiology remains unclear. Annexins are structurally related calcium dependent phospholipid binding proteins with ability to promote contact between vesicle membranes [54]. The proteomic lists contained annexin VI (common to the MTJ and M samples) and annexin I (unique to the MTJ sample). Annexin VI has been involved in the maintenance of the cytoskeleton and extracellular matrix integrity [55] and appears to co-localize with α-actinin at z-discs in cardiomyocytes [56]. Annexin I is expressed in satellite cells of adult muscle [57], but a function in muscle fibre has not been reported. Immunofluorescence with Annexin I antibodies showed very high expression at the MTJ of adult muscle. Given that annexin I has been shown to interact with profilin [55], a crucial protein in the actin polymerization process, it is plausible that annexin I contributes to cytoskeletal remodeling at this localization. αB crystallin is a member of the small heat shock protein family [58] and acts as a molecular chaperone. In skeletal muscle, αB crystallin localizes to the z-disc and interacts with desmin, vimentin, and actin [59,60]. Mutations in αB crystallin underlie myofibrillar myopathies and, crucially, the R120G mutation [61] has been shown to elevate autophagy in a transgenic mouse model [62].

As a tension bearer structure, misfolding of cytoskeletal crosslinkers and associated proteins might occur at the MTJ at higher rates than elsewhere in the fibre. Chaperon mediated degradation of damaged proteins has been linked to increased autophagy and protein turnover [43]. In addition to αB crystallin, several chaperons were identified in the MTJ sample including Hspa8 (Hsc70). Given that Hspa8 has been shown to be involved in the chaperon mediated degradation of filamin C [43], it is plausible that the presence of αB crystallin and other chaperons in the MTJ sample relates to higher protein turnover in this localization. In conclusion, although purely descriptive, our results indicate that LCM and LC/MS-MS of myotendinous junction sections is a plausible experimental approach to identify novel factors involved in tension sensing and their regulation.

## Methods

### Tissue processing

Hind- and fore-limb muscles were dissected from mice and embedded into O.C.T. within small plastic biopsy molds (Sakura Finetek). The whole piece was immediately snap frozen in cold isopentane and stored at −80°C. Catapulting tests (see below) showed that sections of 20 μm were optimal for LCM purification. Sections were placed on polyethylene naphthalate membrane slides (Leica Microsystems). The presence of myotendinous junction was checked every 3 slides, each slide containing 12 to 18 sections. A slight modification of the Hematoxylin and Gurr Eosin protocol (Santa Cruz) was used to stain the slides and facilitate the identification of the myotendionous junctions. Slides were incubated on hematoxylin for 1 minute and then washed under running water for 5 minutes. Eosin was then added just for 10 seconds, followed by a 5 minutes wash as before. Slides were then allowed to dry on an air cabinet overnight. Stained slides were used for LCM.

### LCM

A laser pressure catapult protocol was applied using a PALM LCM inverted microscope (Zeiss). Membrane regions were marked and cut out by a focused laser beam by automated movement along a fixed laser focus. The dissected segments were then catapulted into an adhesive cap of a collection tube by a laser pulse. The volume of energy and focus was adjusted for the complete sectioning of myotendinous and extra junction membrane regions was: collagen rich myotendinous regions required 80% energy and 45% focus while extra junctional regions worked better with 75% energy and 42% focus. These values were also modified as required. Collected pieces were pelleted by brief centrifugation at 14000 *g* and kept at −80°C. All collected samples were pooled into single myotendinous junction and extrajunctional membrane pellets, resuspended into NuPAGE LDS sample buffer, heated at 70°C for 10 minutes and run on a Bis-Tris mini gel (Life technologies), before staining with Bio-Safe Coomassie stain (Bio-Rad).

### LC-MS/MS

Gel sections were selected and excised. To minimise the potential for suppression from high abundance components; intensely stained bands were isolated from regions of low staining as shown in Figure 1B. In-gel tryptic digestion was performed after reduction with DTE and S-carbamidomethylation with iodoacetamide. Gel pieces were washed twice with 50% (v:v) aqueous acetonitrile containing 25 mM ammonium bicarbonate, then once with acetonitrile and dried in a vacuum concentrator for 20 min. Sequencing-grade, modified porcine trypsin (Promega) was dissolved in the 50 mM acetic acid supplied by the manufacturer, then diluted 5-fold by adding 25 mM ammonium bicarbonate to give a final trypsin concentration of 0.02 mg/mL. Gel pieces were rehydrated by adding 10 μL of trypsin solution, and after 30 min enough 25 mM ammonium bicarbonate solution was added to cover the gel pieces. Digests were incubated overnight at 37°C. Peptides were extracted from the gel by washing three times with 50% (v:v) aqueous acetonitrile containing 0.1% trifluoroacetic acid (v:v), before being dried down in a vacuum concentrator and reconstituting in aqueous 0.1% (v:v) trifluoroacetic acid. Each section was digested and analysed by LC-MS/MS independently.

Samples were loaded onto a nanoAcquity UPLC system (Waters) equipped with a nanoAcquity Symmetry C_18_, 5 μm trap (180 μm × 20 mm Waters) and a nanoAcquity BEH130 1.7 μm C_18_ capillary column (75 μm × 250 mm, Waters). The trap wash solvent was 0.1% (v/v) aqueous formic acid and the trapping flow rate was 10 μL/min. The trap was washed for 5 min before switching flow to the capillary column. The separation used a gradient elution of two solvents (solvent A: 0.1% (v/v) formic acid; solvent B: acetonitrile containing 0.1% (v/v) formic acid). The flow rate for the capillary column was 300 nL/min Column temperature was 60°C and the gradient profile was as follows: initial conditions 5% solvent B, followed by a linear gradient to 30% solvent B over 125 min, then a linear gradient to 50% solvent B over 5 min, followed by a wash with 95% solvent B for 10 min. The column was returned to initial conditions and re-equilibrated for 30 min before subsequent injections.

The nanoLC system was interfaced with a maXis LC-MS/MS System (Bruker Daltonics) with a Bruker nano-electrospray source fitted with a steel emitter needle (180 μm O.D. × 30 μm I.D., Thermo (Proxeon)). Positive ESI- MS & MS/MS spectra were acquired using AutoMSMS mode. Instrument control, data acquisition and processing were performed using Compass 1.3 SR3 software (microTOF control, Hystar and DataAnalysis, Bruker Daltonics). Instrument settings were: ion spray voltage: 1,400 V, dry gas: 4 L/min, dry gas temperature 160°C, ion acquisition range: *m/z* 50–2,200. AutoMSMS settings were: MS: 0.5 s (acquisition of survey spectrum), MS/MS (CID with N_2_ as collision gas): ion acquisition range: *m/z* 300–1,500, 0.1 s acquisition for precursor intensities above 100,000 counts, for signals of lower intensities down to 1,000 counts acquisition time increased linear to 1 s, the collision energy and isolation width settings were automatically calculated using the AutoMSMS fragmentation table: 8 precursor ions, absolute threshold 1,000 counts, preferred charge states: 2 – 4, singly charged ions excluded. 1 MS/MS spectrum was acquired for each precursor and former target ions were excluded for 30 s.

### Database search

Spectra were calibrated using a lock mass signal (m/z 1221.99064) prior to compound detection and peak list creation. The peak list files obtained from individual gel sections were combined and then submitted for database searching to a locally-running copy of the Mascot program (Matrix Science Ltd., version 2.3.02), through the ProteinScape interface (Bruker Daltonics., version 2.1). The database searched was IPI.mouse (v3.87 27/11/2011). Search criteria included: enzyme, trypsin; missed cleavages, 1; fixed modifications, carbamidomethyl (C); variable modifications, acetyl (N-terminal) and oxidation (M); peptide tolerance, 10 ppm; MS/MS tolerance, 0.1 Da. The search included an automatic decoy database search and the false discovery rate for identity was <2%. The significance threshold was p < 0.05 and the peptide ion score cut-off was 20.

### Immunofluorescence

Tissues were obtained from 30 to 45 days old C57BL/6 male mice killed by cervical dislocation. Tissues were frozen in isopentane cooled in liquid nitrogen prior to cryosectioning and then stored at −80°C. After thawing and drying, sections were fixed with either cold acetone (pure or as a 1:1 mix with cold methanol) or 4% paraformaldehyde for 30 min and rinsed with PBS three times or used unfixed. Standard indirect immunohistochemistry was then employed using primary antibodies followed by FITC conjugated polyclonal anti-mouse, −rabbit or -goat secondary antibodies using dilutions as recommended by the manufacturer (Sigma-Aldrich). The primary antibodies used in this work were: goat anti human FHL1 (cat. AHP2070, AbD Serotec), mouse monoclonal anti-syntrophin (cat. SAB4200213, Sigma-Aldrich), mouse monoclonal anti-myomesin (cat. mMaC myomesin B4, DSHB), mouse monoclonal anti-titin (cat. 9 D10, DSHB), mouse monoclonal anti s-laminin (cat. C4, DSHB), mouse monoclonal anti filaminC RR90 (an IgA sub-type [31]), mouse monoclonal anti-Annexin I (cat. EH17a, DSHB), mouse monoclonal anti-myotilin (RSO34, Novocastra), mouse monoclonal anti-desmin (cat. D76, DSHB), rabbit polyclonal anti-TACP (cat. QC18385, Sigma-Aldrich), mouse monoclonal anti-αB crystallin (cat. CPTC-CRYAB-3, DSHB), mouse monoclonal anti-tubulin (cat. E7, DSHB), mouse monoclonal anti-α actinin (cat EA53, Sigma-Aldrich), mouse monoclonal anti-α-sarcoglycan (cat. IVD3(1)A9, DSHB). Initial dilutions were at 1:200 in all cases and adjusted accordingly depending on the signal to noise. At the dilutions used, none of the secondary antibodies used on their own gave a detectable signal using identical incubation conditions and confocal settings (data not shown). All images were taken with a Zeiss 510 upright confocal microscope using a plan-Apochromat 63×/1.4 Oil DIC lense at 1024×1024 resolution. Detailed views of regions of interest were obtained using various zoom factors as required, using the same resolution. ImageJ (http://imagej.nih.gov/ij/index.html) was used to merge channels and produce the figures.

## Abbreviations

MTJ: (myotendinous junction); M: (extra junctional membrane); LCM: (laser capture microscopy); LC/MS-MS: (liquid chromatography tandem mass spectrometry); GO: (gene ontology); DAPC: (dystrophin-associated protein complex).

## Competing interests

The authors declare that they have no competing interests.

## Authors’ contributions

TC carried our laser dissections from sections; LF carried out histological preparations and immunostanings; DA carried out LCM/MS/MS; AD carried out SDS gel processing and trypsin digestions; JT provided advice on methodology and literature review; PO provided confocal support for revised manuscript; GB design the experimental plan and wrote the article. All authors read and approved the final manuscript.

## Supplementary Material

Additional file 1: Table S1 Mouse proteins identified in myotendinous junction (MTJ) and peripheral membrane (M) LCM samples. Individual lists are ranked by Mascot score. The ‘Rank’ entry refers to protein’s ranking in the original Mascot results for each sample. Click here for file

Additional file 2: Figure S1 Blind analysis of the most likely cellular components associated with the proteins identified by LC-MS/MS in the myotendinous junction. A diagram generated by the GOrilla software (see text for details) using all proteins identified in the myotendinous junction sample. The color of the box indicates *P* value interval of the enclosed term: white, yellow, orange, brown and red denotes *P* values > 10^−3^, 10^−3^ to 10^−5^, 10^−5^ to 10^−7^,10^−7^ to 10^−9^ and < 10^−9^ respectively. The *P* value is the enrichment p-value computed according to a minimum hypergeometric model and is not corrected for multiple testing (see Additional file 3: Table S2 for corrected *P* values). Click here for file

Additional file 3: Table S2 Full lists of genes per enriched GO term with the corrected p values* for multiple testing [30]. Click here for file
