# Supplementary material for: Proteomic analysis of laser capture microscopy purified myotendinous junction regions from muscle sections
Source: Proteome Sci. 2014 May 7;12:25. doi: 10.1186/1477-5956-12-25 (PMC4113200; doi:10.1186/1477-5956-12-25)
Supplement: Additional file 1: Table S1 — Mouse proteins identified in myotendinous junction (MTJ) and peripheral membrane (M) LCM samples. Individual lists are ranked by Mascot score. The ‘Rank’ entry refers to protein’s ranking in the original Mascot results for each sample. [file 1477-5956-12-25-S1.docx]

# Table S1

Mouse proteins identified in myotendinous junction (MTJ) and peripheral membrane (M) LCM samples. Individual lists are ranked by Mascot score. The ‘Rank’ entry refers to protein’s ranking in the original Mascot results for each sample.

# Identified in MTJ only

| **Protein description** | **Accession** | **Rank** | **Mascot**  **score** | **Number of peptide matches** | **Number of peptide sequences** | **Sequence coverage (%)** |
| --- | --- | --- | --- | --- | --- | --- |
| Ttn Uncharacterized protein | IPI00986455 | 3 | 19546 | 576 | 492 | 21.8 |
| Myl1 Isoform MLC3 of Myosin light chain 1/3, skeletal muscle isoform | IPI00469531 | 14 | 3239 | 96 | 14 | 92.7 |
| Eno1;LOC100503183;LOC100045967;Gm5506 Alpha-enolase | IPI00462072 | 41 | 1212 | 31 | 7 | 24.7 |
| 4732456N10Rik hypothetical protein LOC239673 | IPI00222228 | 60 | 785 | 28 | 7 | 11.2 |
| Myl3 Myosin light chain 3 | IPI00133392 | 67 | 680 | 27 | 6 | 42.6 |
| Tnnt3 Isoform A5e17 of Troponin T, fast skeletal muscle | IPI00229055 | 73 | 532 | 13 | 5 | 27.4 |
| Jup Junction plakoglobin | IPI00229475 | 97 | 273 | 6 | 5 | 9.9 |
| Plec plectin isoform 1b2alpha | IPI00229509 | 100 | 249 | 6 | 6 | 2.3 |
| Eef1a1 Elongation factor 1-alpha 1 | IPI00307837 | 109 | 201 | 9 | 5 | 14.5 |
| Ogdh Isoform 4 of 2-oxoglutarate dehydrogenase, mitochondrial | IPI00420882 | 116 | 189 | 5 | 5 | 7.3 |
| Myl2 Myosin regulatory light chain 2, ventricular/cardiac muscle isoform | IPI00555015 | 135 | 120 | 4 | 2 | 18.7 |
| Ryr1 ryanodine receptor 1, skeletal muscle | IPI00622912 | 138 | 112 | 5 | 4 | 1.2 |
| Gys1 Glycogen [starch] synthase, muscle | IPI00130127 | 144 | 102 | 2 | 2 | 4.7 |
| Mpz myelin protein P0 precursor | IPI01008333 | 147 | 102 | 3 | 2 | 9.3 |
| Ahsg Alpha-2-HS-glycoprotein | IPI00128249 | 153 | 91 | 1 | 1 | 7.0 |
| Anxa6 annexin A6 isoform b | IPI00310240 | 157 | 89 | 3 | 3 | 5.7 |
| Obscn obscurin isoform 1 | IPI00459500 | 158 | 84 | 2 | 2 | 0.4 |
| Fabp5 Fatty acid-binding protein, epidermal | IPI00114162 | 162 | 80 | 1 | 1 | 6.7 |
| Cpt1b Carnitine O-palmitoyltransferase 1, muscle isoform | IPI00136563 | 167 | 80 | 1 | 1 | 1.6 |
| Atl2 Uncharacterized protein | IPI00227199 | 180 | 58 | 1 | 1 | 3.6 |
| Cox4i1 Cytochrome c oxidase subunit 4 isoform 1, mitochondrial | IPI00117978 | 191 | 45 | 2 | 2 | 13.0 |
| Ddx25 Isoform 1 of ATP-dependent RNA helicase DDX25 | IPI00135420 | 206 | 28 | 2 | 1 | 5.2 |
| Anxa1 Annexin A1 | IPI00230395 | 213 | 24 | 1 | 1 | 3.2 |
| Lyz1 Lysozyme C-1 | IPI00113427 | 215 | 24 | 1 | 1 | 8.1 |
| LOC100046079;Gm11273;Cox5b cytochrome c oxidase subunit 5B, mitochondrial | IPI00116154 | 216 | 24 | 1 | 1 | 9.3 |
| Bdh1 D-beta-hydroxybutyrate dehydrogenase, mitochondrial | IPI00330754 | 218 | 24 | 1 | 1 | 2.0 |
| Dsg1c Isoform 1 of Desmoglein-1-gamma | IPI00331259 | 219 | 24 | 1 | 1 | 1.1 |
| Lrpprc Leucine-rich PPR motif-containing protein, mitochondrial | IPI00420706 | 223 | 23 | 1 | 1 | 0.7 |
| 1700071K01Rik RIKEN cDNA 1700071K01 gene | IPI00126917 | 226 | 22 | 1 | 1 | 3.7 |
| Acadm Medium-chain specific acyl-CoA dehydrogenase, mitochondrial | IPI00134961 | 228 | 21 | 2 | 2 | 5.9 |
| Serpinb6b Uncharacterized protein | IPI00116247 | 230 | 20 | 3 | 1 | 4.0 |
| Lmna Isoform C2 of Prelamin-A/C | IPI00230435 | 236 | 17 | 1 | 1 | 1.9 |
| Tubb4 Tubulin beta-4 chain | IPI00109073 | 240 | 17 | 1 | 1 | 3.4 |
| Tbc1d5 TBC1 domain family member 5 | IPI00119109 | 241 | 17 | 1 | 1 | 2.0 |
| Dclre1c Isoform 1 of Protein artemis | IPI00170222 | 244 | 17 | 1 | 1 | 1.8 |
| Gfm2 Isoform 3 of Ribosome-releasing factor 2, mitochondrial | IPI00269240 | 246 | 17 | 1 | 1 | 2.5 |
| Eprs Bifunctional aminoacyl-tRNA synthetase | IPI00339916 | 249 | 17 | 1 | 1 | 0.8 |
| Cfl1 Putative uncharacterized protein | IPI00407543 | 250 | 17 | 1 | 1 | 5.2 |
| Sae1 Isoform 1 of SUMO-activating enzyme subunit 1 | IPI00129105 | 255 | 17 | 1 | 1 | 3.1 |
| Uba1 Ubiquitin-like modifier-activating enzyme 1 | IPI00123313 | 256 | 17 | 1 | 1 | 2.0 |
| Ndufa6 NADH dehydrogenase [ubiquinone] 1 alpha subcomplex subunit 6 | IPI00133399 | 258 | 17 | 1 | 1 | 6.1 |
| Gm6866 uncharacterized protein C2orf78 homolog | IPI00989535 | 261 | 17 | 1 | 1 | 1.6 |
| Rfx3 Putative uncharacterized protein (Fragment) | IPI00653945 | 262 | 17 | 1 | 1 | 1.1 |
| Shpk Sedoheptulokinase | IPI00108042 | 263 | 17 | 1 | 1 | 2.7 |

# Identified in M only

| **Protein description** | **Accession** | **Rank** | **Mascot score** | **Number of peptide matches** | **Number of peptide sequences** | **Sequence coverage (%)** |
| --- | --- | --- | --- | --- | --- | --- |
| Actc1 Actin, alpha cardiac muscle 1 | IPI00114593 | 4 | 19409 | 521 | 21 | 66.3 |
| Myl1 Uncharacterized protein | IPI00461505 | 11 | 5415 | 151 | 13 | 69.6 |
| Myh13 Uncharacterized protein | IPI00468665 | 17 | 4144 | 147 | 38 | 19.2 |
| LOC100503183 alpha-enolase-like isoform 11 | IPI00990455 | 29 | 2091 | 47 | 10 | 34.3 |
| Ttn Isoform 1 of Titin | IPI00756257 | 39 | 1369 | 70 | 69 | 3.1 |
| Dcn Decorin | IPI00123196 | 46 | 1172 | 41 | 13 | 36.7 |
| Tnnt3 Isoform B2e17 of Troponin T, fast skeletal muscle | IPI00229058 | 52 | 1006 | 25 | 9 | 44.0 |
| Hbb-b2 Hemoglobin subunit beta-2 | IPI00316491 | 70 | 631 | 28 | 6 | 44.2 |
| Lum Lumican | IPI00313900 | 82 | 481 | 14 | 7 | 25.4 |
| Serpina3k Serine protease inhibitor A3K | IPI00131830 | 85 | 468 | 13 | 8 | 25.1 |
| Tubb2c Tubulin beta-2C chain | IPI00169463 | 88 | 415 | 19 | 13 | 38.2 |
| Hspa5 78 kDa glucose-regulated protein | IPI00319992 | 99 | 358 | 8 | 5 | 8.7 |
| Hist1h2bj;Hist1h2bn;Hist1h2bf;Hist1h2bl Histone H2B type 1-F/J/L | IPI00114642 | 117 | 252 | 9 | 3 | 26.2 |
| Spr sepiapterin reductase | IPI00129164 | 125 | 203 | 2 | 2 | 8.8 |
| Phb Prohibitin | IPI00133440 | 132 | 191 | 7 | 6 | 27.2 |
| Kera Putative uncharacterized protein | IPI00129867 | 133 | 191 | 10 | 6 | 18.7 |
| Anxa6 Annexin A6 | IPI00554894 | 134 | 181 | 10 | 6 | 12.5 |
| Serpina1d Alpha-1-antitrypsin 1-4 | IPI00123924 | 136 | 170 | 16 | 5 | 16.2 |
| Serpina3m Serine protease inhibitor A3M | IPI00135635 | 141 | 155 | 6 | 5 | 14.8 |
| Hpx Hemopexin | IPI00128484 | 143 | 153 | 4 | 3 | 9.1 |
| Serpinf1 Pigment epithelium-derived factor | IPI00331088 | 144 | 153 | 6 | 6 | 22.5 |
| Prdx1 Peroxiredoxin-1 | IPI00121788 | 145 | 152 | 5 | 5 | 24.1 |
| Gpx3 Glutathione peroxidase 3 | IPI00133536 | 147 | 151 | 3 | 2 | 11.5 |
| Myh9 Myosin-9 | IPI00123181 | 148 | 146 | 4 | 2 | 1.0 |
| Gc Vitamin D-binding protein | IPI00126184 | 157 | 128 | 2 | 2 | 6.5 |
| Mcpt4 mast cell protease 4 precursor | IPI00788456 | 159 | 124 | 3 | 3 | 14.6 |
| Lmna Isoform C of Prelamin-A/C | IPI00400300 | 164 | 116 | 4 | 4 | 8.2 |
| Cpa3 Mast cell carboxypeptidase A | IPI00126316 | 165 | 113 | 2 | 2 | 5.5 |
| Adsl Adenylosuccinate lyase | IPI00308217 | 166 | 113 | 2 | 2 | 4.5 |
| Eif4a1 Eukaryotic initiation factor 4A-I | IPI00118676 | 167 | 110 | 2 | 1 | 2.5 |
| Cav1 Isoform Alpha of Caveolin-1 | IPI00117829 | 168 | 108 | 1 | 1 | 7.9 |
| Sar1a Putative uncharacterized protein | IPI00115644 | 170 | 108 | 1 | 1 | 5.6 |
| Fgb Fibrinogen beta chain | IPI00279079 | 171 | 108 | 1 | 1 | 3.5 |
| Ppp2r1a Serine/threonine-protein phosphatase 2A 65 kDa regulatory subunit A alpha isoform | IPI00310091 | 172 | 108 | 1 | 1 | 3.4 |
| Apoa1 Apolipoprotein A-I | IPI00121209 | 174 | 105 | 5 | 3 | 12.5 |
| Tuba8 Tubulin alpha-8 chain | IPI00311175 | 176 | 102 | 7 | 4 | 13.8 |
| Cyc1 Isoform 1 of Cytochrome c1, heme protein, mitochondrial | IPI00132728 | 178 | 99 | 9 | 5 | 23.1 |
| Ywhaz 14-3-3 protein zeta/delta | IPI00116498 | 184 | 92 | 5 | 3 | 13.5 |
| Fhl3 Uncharacterized protein | IPI00828338 | 185 | 83 | 1 | 1 | 6.6 |
| Park7 Protein DJ-1 | IPI00117264 | 189 | 76 | 8 | 7 | 43.4 |
| Aldh2 Aldehyde dehydrogenase, mitochondrial | IPI00111218 | 198 | 64 | 3 | 3 | 8.5 |
| Ndufs2 NADH dehydrogenase [ubiquinone] iron-sulfur protein 2, mitochondrial | IPI00128023 | 200 | 63 | 3 | 2 | 4.3 |
| Ndufa13 NADH dehydrogenase [ubiquinone] 1 alpha subcomplex subunit 13 | IPI00230715 | 204 | 61 | 2 | 2 | 13.2 |
| Capzb Isoform 1 of F-actin-capping protein subunit beta | IPI00406800 | 205 | 60 | 4 | 2 | 8.3 |
| Pzp Uncharacterized protein | IPI00624663 | 206 | 59 | 4 | 4 | 2.9 |
| Ywhab Isoform Long of 14-3-3 protein beta/alpha | IPI00230682 | 207 | 57 | 5 | 3 | 12.2 |
| Ppp1cb Serine/threonine-protein phosphatase PP1-beta catalytic subunit | IPI00311873 | 208 | 56 | 2 | 1 | 4.0 |
| Cacnb1 Isoform 1 of Voltage-dependent L-type calcium channel subunit beta-1 | IPI00321850 | 209 | 56 | 2 | 2 | 5.4 |
| Rap1a Ras-related protein Rap-1A | IPI00138406 | 211 | 54 | 2 | 1 | 6.5 |
| Cacna2d1 Isoform 2B of Voltage-dependent calcium channel subunit alpha-2/delta-1 | IPI00230013 | 212 | 54 | 5 | 4 | 5.4 |
| Fgg Uncharacterized protein | IPI00122312 | 216 | 51 | 3 | 3 | 6.8 |
| Eef1d Isoform 1 of Elongation factor 1-delta | IPI00118875 | 218 | 51 | 2 | 2 | 8.5 |
| Ndufa4 NADH dehydrogenase [ubiquinone] 1 alpha subcomplex subunit 4 | IPI00125929 | 223 | 50 | 3 | 2 | 24.4 |
| Ank1 Isoform Mu7 of Ankyrin-1 | IPI00109307 | 224 | 49 | 1 | 1 | 7.7 |
| Macrod1 MACRO domain-containing protein 1 | IPI00122740 | 225 | 49 | 1 | 1 | 7.1 |
| Synpo2 Isoform 1 of Synaptopodin-2 | IPI00130162 | 226 | 49 | 1 | 1 | 1.7 |
| Psmc5 26S protease regulatory subunit 8 | IPI00135640 | 227 | 49 | 1 | 1 | 3.2 |
| Lrig1 Leucine-rich repeats and immunoglobulin-like domains protein 1 | IPI00136475 | 228 | 49 | 1 | 1 | 2.0 |
| Pdia6 Putative uncharacterized protein | IPI00222496 | 229 | 49 | 1 | 1 | 3.8 |
| Ftl1 ferritin light chain 1 | IPI00608020 | 236 | 45 | 3 | 2 | 21.3 |
| Aspn Asporin | IPI00117957 | 237 | 45 | 3 | 1 | 2.9 |
| Pdlim7 Isoform 2 of PDZ and LIM domain protein 7 | IPI00315878 | 242 | 38 | 3 | 2 | 11.2 |
| Tpm4 Tropomyosin alpha-4 chain | IPI00421223 | 245 | 36 | 5 | 3 | 11.3 |
| Fabp3 Fatty acid-binding protein, heart | IPI00230124 | 247 | 36 | 3 | 3 | 24.1 |
| Bin1 Isoform 1 of Myc box-dependent-interacting protein 1 | IPI00114352 | 250 | 33 | 3 | 3 | 8.8 |
| Psmb6 Proteasome subunit beta type-6 | IPI00119239 | 251 | 33 | 1 | 1 | 4.6 |
| Pfn1 Profilin-1 | IPI00224740 | 252 | 33 | 1 | 1 | 11.4 |
| Camk2a Isoform Alpha KAP of Calcium/calmodulin-dependent protein kinase type II subunit alpha | IPI00230096 | 253 | 33 | 1 | 1 | 6.0 |
| Fkbp1a Peptidyl-prolyl cis-trans isomerase FKBP1A | IPI00266899 | 254 | 33 | 1 | 1 | 12.0 |
| Rplp0 60S acidic ribosomal protein P0 | IPI00314950 | 255 | 33 | 1 | 1 | 9.8 |
| Arhgdia Rho GDP-dissociation inhibitor 1 | IPI00322312 | 256 | 33 | 1 | 1 | 7.8 |
| 6030445D17Rik Putative uncharacterized protein (Fragment) | IPI00228532 | 259 | 32 | 5 | 1 | 5.0 |
| Hnrnpa2b1 Isoform 3 of Heterogeneous nuclear ribonucleoproteins A2/B1 | IPI00405058 | 260 | 32 | 1 | 1 | 3.3 |
| Cdh13 Cadherin-13 | IPI00123746 | 262 | 31 | 2 | 2 | 3.6 |
| Aldh6a1 Methylmalonate-semialdehyde dehydrogenase [acylating], mitochondrial | IPI00461964 | 263 | 31 | 2 | 2 | 5.8 |
| Prkar2a cAMP-dependent protein kinase type II-alpha regulatory subunit | IPI00116546 | 264 | 30 | 1 | 1 | 3.7 |
| Prkaca Isoform 2 of cAMP-dependent protein kinase catalytic subunit alpha | IPI00227900 | 265 | 28 | 2 | 1 | 3.5 |
| Hspa9 Stress-70 protein, mitochondrial | IPI00133903 | 266 | 27 | 2 | 2 | 4.0 |
| Calm2;Calm1;Calm3 Calmodulin | IPI00761696 | 268 | 26 | 2 | 2 | 13.4 |
| Ndufa2 NADH dehydrogenase [ubiquinone] 1 alpha subcomplex subunit 2 | IPI00315302 | 269 | 26 | 2 | 1 | 14.1 |
| Snta1 alpha-1-syntrophin | IPI00751974 | 271 | 26 | 1 | 1 | 1.6 |
| Serpinb6a Serpin B6 | IPI00121471 | 275 | 25 | 1 | 1 | 2.1 |
| Ndufs4 NADH dehydrogenase [ubiquinone] iron-sulfur protein 4, mitochondrial | IPI00229008 | 276 | 25 | 2 | 1 | 8.6 |
| Acp1;LOC631286 Isoform 1 of Low molecular weight phosphotyrosine protein phosphatase | IPI00134135 | 277 | 24 | 1 | 1 | 5.7 |
| Gm5409 Try10-like trypsinogen | IPI00395100 | 278 | 24 | 2 | 1 | 8.1 |
| 1810009J06Rik RIKEN cDNA 1810009J06 | IPI00131673 | 281 | 24 | 1 | 1 | 8.1 |
| Psmd6 26S proteasome non-ATPase regulatory subunit 6 | IPI00319965 | 282 | 24 | 1 | 1 | 3.9 |
| Pgp Phosphoglycolate phosphatase | IPI00380195 | 283 | 24 | 1 | 1 | 4.7 |
| Idh3b isocitrate dehydrogenase 3, beta subunit | IPI00126635 | 284 | 24 | 1 | 1 | 2.3 |
| Echs1 Enoyl-CoA hydratase, mitochondrial | IPI00454049 | 285 | 24 | 1 | 1 | 4.5 |
| Gyg Glycogenin-1 | IPI00264062 | 286 | 24 | 3 | 1 | 4.8 |
| Anxa11 Annexin A11 | IPI00124264 | 287 | 23 | 1 | 1 | 1.8 |
| Rps3 40S ribosomal protein S3 | IPI00134599 | 288 | 23 | 1 | 1 | 3.7 |
| C3 Isoform Long of Complement C3 (Fragment) | IPI00323624 | 289 | 23 | 1 | 1 | 0.8 |
| Uqcrfs1 Cytochrome b-c1 complex subunit Rieske, mitochondrial | IPI00133240 | 290 | 23 | 3 | 3 | 10.6 |
| Gpd2 Uncharacterized protein | IPI00331182 | 291 | 23 | 2 | 2 | 3.8 |
| Zbtb8os Uncharacterized protein | IPI00880296 | 293 | 22 | 2 | 1 | 14.8 |
| Psma8 Proteasome subunit alpha type-7-like | IPI00109122 | 296 | 21 | 2 | 2 | 10.0 |
| Hsp90b1 Endoplasmin | IPI00129526 | 297 | 21 | 2 | 2 | 3.2 |
| Gbas Protein NipSnap homolog 2 | IPI00115827 | 298 | 20 | 2 | 2 | 7.5 |
| Adipoq Adiponectin | IPI00311383 | 300 | 19 | 2 | 2 | 7.7 |
| Rab1b Ras-related protein Rab-1B | IPI00133706 | 301 | 19 | 1 | 1 | 8.0 |
| Cox6c Cytochrome c oxidase subunit 6C | IPI00131771 | 305 | 18 | 2 | 1 | 15.8 |
| Patl2 Protein PAT1 homolog 2 | IPI00108109 | 306 | 18 | 1 | 1 | 3.0 |
| Serpinh1 Serpin H1 | IPI00114733 | 307 | 18 | 1 | 1 | 2.9 |
| Kng1 Isoform HMW of Kininogen-1 | IPI00114958 | 308 | 18 | 1 | 1 | 1.5 |
| Fis1 Mitochondrial fission 1 protein | IPI00132217 | 309 | 18 | 1 | 1 | 9.9 |
| Pde8b phosphodiesterase 8B isoform 1 | IPI00221980 | 311 | 18 | 1 | 1 | 0.8 |
| Cilp2 cartilage intermediate layer protein 2 | IPI00282930 | 312 | 18 | 1 | 1 | 1.3 |
| Bank1 B-cell scaffold protein with ankyrin repeats | IPI00330444 | 314 | 18 | 1 | 1 | 1.1 |
| Lztfl1 Leucine zipper transcription factor-like protein 1 | IPI00458573 | 317 | 18 | 1 | 1 | 3.7 |
| Ppia Peptidyl-prolyl cis-trans isomerase | IPI00554989 | 319 | 18 | 1 | 1 | 10.8 |
| Megf8 Multiple epidermal growth factor-like domains protein 8 | IPI00930841 | 320 | 18 | 1 | 1 | 0.2 |
| Rpl11 60S ribosomal protein L11 | IPI00331461 | 321 | 17 | 1 | 1 | 7.9 |
| Ankib1 Ankib1 protein | IPI00467355 | 322 | 17 | 1 | 1 | 3.2 |
| Polr1d;Gm12751 Polymerase (RNA) I polypeptide D | IPI00480494 | 323 | 17 | 1 | 1 | 5.6 |
| Ctsd Cathepsin D | IPI00111013 | 325 | 16 | 1 | 1 | 5.4 |
| Hnrnpk Isoform 2 of Heterogeneous nuclear ribonucleoprotein K | IPI00224575 | 331 | 16 | 1 | 1 | 5.0 |
| Gprin2 Uncharacterized protein | IPI00750996 | 332 | 15 | 2 | 1 | 2.0 |
| Lamc1 Laminin subunit gamma-1 | IPI00400016 | 333 | 15 | 1 | 1 | 0.9 |
| Vapb Vesicle-associated membrane protein-associated protein B | IPI00135655 | 334 | 15 | 2 | 2 | 9.9 |
| Smarcd2 Isoform 2 of SWI/SNF-related matrix-associated actin-dependent regulator of chromatin subfamily D member 2 | IPI00115461 | 335 | 14 | 1 | 1 | 1.7 |
| Mtap2 Microtubule-associated protein 2 | IPI00118075 | 336 | 14 | 1 | 1 | 1.0 |
| Lrp1b Uncharacterized protein | IPI00119787 | 337 | 14 | 1 | 1 | 0.3 |
| Apoo Apolipoprotein O | IPI00121576 | 338 | 14 | 1 | 1 | 2.8 |
| Mug1 Murinoglobulin-1 | IPI00123223 | 339 | 14 | 1 | 1 | 0.7 |
| Aifm1 Apoptosis-inducing factor 1, mitochondrial | IPI00129577 | 340 | 14 | 1 | 1 | 1.8 |
| Pgls 6-phosphogluconolactonase | IPI00132080 | 341 | 14 | 1 | 1 | 10.1 |
| Mga MAX gene-associated protein isoform 1 | IPI00135072 | 342 | 14 | 1 | 1 | 0.5 |
| Slc26a8 Isoform 2 of Testis anion transporter 1 | IPI00153170 | 343 | 14 | 1 | 1 | 3.1 |
| Gabbr1 Isoform 1A of Gamma-aminobutyric acid type B receptor subunit 1 | IPI00223325 | 344 | 14 | 1 | 1 | 0.9 |
| Kifc2 Kinesin-like protein KIFC2 | IPI00309091 | 346 | 14 | 1 | 1 | 1.5 |
| 2410127L17Rik UPF0586 protein C9orf41 homolog | IPI00330404 | 347 | 14 | 1 | 1 | 3.2 |
| Xpo4 Exportin-4 | IPI00331063 | 348 | 14 | 1 | 1 | 3.0 |
| Prdx6 Putative uncharacterized protein | IPI00404031 | 350 | 14 | 1 | 1 | 6.2 |
| Vps13d Vacuolar protein sorting 13D | IPI00649141 | 351 | 14 | 1 | 1 | 0.2 |
| Dmxl2 Isoform 3 of DmX-like protein 2 | IPI00853917 | 352 | 14 | 1 | 1 | 1.6 |
| Cacna1i Uncharacterized protein | IPI00970640 | 353 | 14 | 1 | 1 | 6.1 |
| Protein Gm20441 | IPI01027554 | 355 | 14 | 1 | 1 | 5.6 |
| Cd36 Platelet glycoprotein 4 | IPI00331214 | 356 | 14 | 1 | 1 | 3.6 |
| Gstm7 Glutathione S-transferase Mu 7 | IPI00121280 | 360 | 14 | 1 | 1 | 7.8 |
| Pdhx Pyruvate dehydrogenase protein X component, mitochondrial | IPI00222767 | 361 | 14 | 1 | 1 | 1.6 |
| Fth1 Ferritin heavy chain | IPI00230145 | 362 | 14 | 1 | 1 | 6.0 |
| Nr3c2 Uncharacterized protein | IPI00918083 | 363 | 14 | 1 | 1 | 11.7 |
| 1700069L16Rik Uncharacterized protein | IPI00918953 | 365 | 14 | 1 | 1 | 24.4 |
| Pgbd5 Uncharacterized protein | IPI00272854 | 366 | 14 | 1 | 1 | 3.1 |
| Fnip2 Uncharacterized protein | IPI00659512 | 368 | 14 | 1 | 1 | 1.1 |
| Ranbp17 Ran-binding protein 17 | IPI00118739 | 369 | 14 | 1 | 1 | 1.2 |
| H2-D1 H-2 class I histocompatibility antigen, D-P alpha chain | IPI00126301 | 370 | 14 | 1 | 1 | 4.3 |
| Piwil1 Isoform 1 of Piwi-like protein 1 | IPI00125402 | 371 | 14 | 1 | 1 | 0.7 |
| Gm13060 zinc finger protein 683-like | IPI00987335 | 372 | 14 | 1 | 1 | 2.4 |
| Serpinc1 Antithrombin-III | IPI00136642 | 373 | 14 | 1 | 1 | 1.9 |
| Chchd3 Coiled-coil-helix-coiled-coil-helix domain-containing protein 3, mitochondrial | IPI00133562 | 374 | 14 | 1 | 1 | 6.6 |
| Ehd2 EH domain-containing protein 2 | IPI00402968 | 375 | 14 | 1 | 1 | 1.7 |
| Sgca Alpha-sarcoglycan | IPI00322911 | 376 | 14 | 1 | 1 | 2.1 |
| Rtn4 Isoform 3 of Reticulon-4 | IPI00120415 | 377 | 14 | 1 | 1 | 7.0 |
| Tmed10 Isoform 1 of Transmembrane emp24 domain-containing protein 10 | IPI00466570 | 380 | 13 | 1 | 1 | 5.0 |
| Crat Carnitine O-acetyltransferase | IPI00113347 | 381 | 13 | 1 | 1 | 1.8 |
| Psmb2 Proteasome subunit beta type-2 | IPI00128945 | 382 | 13 | 1 | 1 | 3.0 |

# Common proteins

|  |  | **Identified in M** | | | | | **Identified in J** | | | | |
| --- | --- | --- | --- | --- | --- | --- | --- | --- | --- | --- | --- |
| **Protein description** | **Accession** | **Rank** | **Mascot score** | **Number of peptide matches** | **Number of peptide sequences** | **Sequence coverage (%)** | **Rank** | **Mascot score** | **Number of peptide matches** | **Number of peptide sequences** | **Sequence coverage (%)** |
| Myh4 Myosin-4 | IPI00404837 | 1 | 23479 | 680 | 108 | 57.3 | 1 | 23788 | 621 | 126 | 62.4 |
| Acta1 Actin, alpha skeletal muscle | IPI00110827 | 2 | 21394 | 572 | 21 | 66.3 | 2 | 20588 | 551 | 123 | 62.4 |
| Myh1 Myosin-1 | IPI00380896 | 3 | 19555 | 570 | 104 | 55.4 | 4 | 19262 | 517 | 117 | 58.2 |
| Myh2 myosin heavy chain IIa | IPI00649292 | 5 | 17036 | 494 | 92 | 48.6 | 5 | 11770 | 334 | 21 | 67.4 |
| Ckm Creatine kinase M-type | IPI00127596 | 6 | 10476 | 294 | 19 | 61.7 | 6 | 8785 | 169 | 17 | 57.2 |
| Actb Actin, cytoplasmic 1 | IPI00110850 | 7 | 9713 | 308 | 17 | 53.3 | 7 | 7633 | 220 | 45 | 51.2 |
| Atp2a1 Sarcoplasmic/endoplasmic reticulum calcium ATPase 1 | IPI00311654 | 8 | 8776 | 259 | 38 | 45.4 | 8 | 5921 | 190 | 13 | 37.6 |
| Aldoa Fructose-bisphosphate aldolase A | IPI00221402 | 9 | 7378 | 218 | 22 | 73.6 | 9 | 4393 | 128 | 21 | 73.9 |
| Myl1 Isoform MLC1 of Myosin light chain 1/3, skeletal muscle isoform | IPI00312700 | 10 | 5697 | 153 | 14 | 68.1 | 10 | 3876 | 101 | 20 | 59.5 |
| Actn3 Alpha-actinin-3 | IPI00136701 | 12 | 4955 | 168 | 39 | 52.8 | 11 | 3790 | 113 | 35 | 51.0 |
| Eno3 Beta-enolase | IPI00228548 | 13 | 4611 | 119 | 20 | 58.5 | 12 | 3515 | 105 | 16 | 84.0 |
| Actbl2 Beta-actin-like protein 2 | IPI00221528 | 14 | 4605 | 145 | 7 | 19.1 | 13 | 3335 | 103 | 15 | 88.8 |
| Alb Serum albumin | IPI00131695 | 15 | 4416 | 170 | 32 | 61.5 | 15 | 3058 | 95 | 6 | 15.4 |
| Mylpf Myosin regulatory light chain 2, skeletal muscle isoform | IPI00224549 | 16 | 4342 | 142 | 17 | 92.9 | 16 | 2807 | 90 | 16 | 46.5 |
| Atp5b ATP synthase subunit beta, mitochondrial | IPI00468481 | 18 | 3475 | 111 | 21 | 58.4 | 17 | 2631 | 90 | 35 | 37.2 |
| Gapdh;LOC100042025 Glyceraldehyde-3-phosphate dehydrogenase | IPI00273646 | 19 | 3454 | 119 | 13 | 52.0 | 18 | 2546 | 72 | 13 | 38.0 |
| Beta-s;Hbb-b1 hemoglobin subunit beta-1-like | IPI00988950 | 20 | 3259 | 94 | 9 | 76.2 | 20 | 2323 | 67 | 12 | 45.6 |
| Atp5a1 ATP synthase subunit alpha, mitochondrial | IPI00130280 | 21 | 3162 | 103 | 21 | 50.6 | 21 | 2301 | 73 | 14 | 38.7 |
| Tpm1 Isoform 1 of Tropomyosin alpha-1 chain | IPI00123316 | 22 | 3144 | 96 | 21 | 53.2 | 24 | 2126 | 65 | 33 | 42.7 |
| Col1a2 Collagen alpha-2(I) chain | IPI00222188 | 23 | 3023 | 91 | 11 | 8.8 | 25 | 2115 | 62 | 16 | 39.4 |
| Mybpc1 myosin binding protein C, slow type | IPI00396774 | 24 | 2983 | 82 | 33 | 35.3 | 26 | 2014 | 85 | 35 | 49.2 |
| Pygm Glycogen phosphorylase, muscle form | IPI00225275 | 25 | 2937 | 114 | 37 | 49.0 | 28 | 1851 | 68 | 63 | 12.4 |
| Tpm2 Isoform 1 of Tropomyosin beta chain | IPI00123319 | 26 | 2804 | 91 | 19 | 53.9 | 29 | 1778 | 53 | 13 | 46.8 |
| Pkm2 Isoform M1 of Pyruvate kinase isozymes M1/M2 | IPI00845840 | 27 | 2744 | 96 | 25 | 54.0 | 31 | 1595 | 37 | 4 | 33.1 |
| Actn2 alpha-actinin-2 | IPI00387557 | 28 | 2681 | 103 | 32 | 49.0 | 32 | 1523 | 44 | 14 | 54.1 |
| Srl Isoform 1 of Sarcalumenin | IPI00224456 | 30 | 1963 | 75 | 18 | 27.3 | 34 | 1399 | 48 | 18 | 46.9 |
| Col6a3 Uncharacterized protein | IPI00845618 | 31 | 1832 | 87 | 40 | 35.7 | 36 | 1332 | 46 | 9 | 8.3 |
| Mybpc2 Myosin-binding protein C, fast-type | IPI00169994 | 33 | 1718 | 61 | 24 | 30.0 | 39 | 1249 | 41 | 9 | 81.6 |
| Mdh2 Malate dehydrogenase, mitochondrial | IPI00323592 | 34 | 1697 | 56 | 16 | 59.8 | 42 | 1190 | 47 | 12 | 48.0 |
| Fmod Fibromodulin | IPI00120187 | 35 | 1487 | 48 | 8 | 30.9 | 45 | 1139 | 39 | 13 | 19.9 |
| Des Desmin | IPI00130102 | 36 | 1462 | 58 | 28 | 60.8 | 46 | 1129 | 33 | 11 | 53.8 |
| Tpm1 tropomyosin alpha-1 chain isoform 2 | IPI00830604 | 38 | 1437 | 49 | 13 | 30.3 | 48 | 1067 | 38 | 8 | 60.4 |
| Tnnc2 Troponin C, skeletal muscle | IPI00284119 | 40 | 1367 | 44 | 9 | 60.0 | 50 | 1041 | 30 | 19 | 17.4 |
| Col1a1 Isoform 1 of Collagen alpha-1(I) chain | IPI00329872 | 41 | 1357 | 45 | 5 | 3.9 | 51 | 1037 | 40 | 10 | 23.2 |
| Ckmt2 Creatine kinase S-type, mitochondrial | IPI00120076 | 42 | 1296 | 53 | 16 | 47.3 | 52 | 1033 | 30 | 11 | 13.5 |
| Tpi1 triosephosphate isomerase | IPI00467833 | 43 | 1285 | 44 | 10 | 49.2 | 53 | 1029 | 30 | 15 | 18.9 |
| Pvalb Parvalbumin alpha | IPI00230766 | 44 | 1215 | 36 | 5 | 48.2 | 54 | 1020 | 45 | 18 | 38.2 |
| Myom2 myomesin 2 | IPI00115823 | 45 | 1187 | 49 | 27 | 25.5 | 55 | 998 | 35 | 16 | 37.3 |
| Casq1 Calsequestrin-1 | IPI00986042 | 47 | 1162 | 37 | 8 | 25.9 | 56 | 898 | 23 | 6 | 46.9 |
| Vdac1 Isoform Mt-VDAC1 of Voltage-dependent anion-selective channel protein 1 | IPI00230540 | 48 | 1135 | 38 | 12 | 59.0 | 59 | 826 | 27 | 7 | 20.5 |
| Slc25a4 ADP/ATP translocase 1 | IPI00115564 | 50 | 1073 | 43 | 10 | 33.6 | 61 | 771 | 33 | 16 | 23.6 |
| Col6a3 Uncharacterized protein (Fragment) | IPI00830749 | 51 | 1012 | 36 | 11 | 17.6 | 62 | 751 | 23 | 20 | 10.2 |
| Actn1 Alpha-actinin-1 | IPI00380436 | 53 | 997 | 32 | 9 | 10.8 | 63 | 727 | 22 | 9 | 34.3 |
| Ldha L-lactate dehydrogenase A chain | IPI00319994 | 54 | 971 | 38 | 11 | 37.0 | 64 | 700 | 23 | 8 | 15.5 |
| Ak1 Isoform 1 of Adenylate kinase isoenzyme 1 | IPI00128209 | 55 | 959 | 35 | 10 | 55.7 | 65 | 693 | 20 | 10 | 27.7 |
| Col6a1 Collagen alpha-1(VI) chain | IPI00339885 | 57 | 861 | 40 | 18 | 21.1 | 66 | 683 | 25 | 16 | 12.9 |
| Mb Myoglobin | IPI00230760 | 59 | 775 | 30 | 5 | 45.5 | 68 | 666 | 27 | 9 | 33.7 |
| Col12a1 Isoform 1 of Collagen alpha-1(XII) chain | IPI00121430 | 60 | 767 | 56 | 37 | 17.7 | 69 | 664 | 24 | 5 | 48.2 |
| Prelp Prolargin | IPI00122293 | 63 | 718 | 29 | 10 | 26.7 | 70 | 624 | 20 | 10 | 23.0 |
| Tnni2 Troponin I, fast skeletal muscle | IPI00223196 | 65 | 702 | 21 | 4 | 20.3 | 71 | 620 | 27 | 12 | 41.9 |
| Vim Vimentin | IPI00227299 | 67 | 681 | 21 | 14 | 29.6 | 72 | 539 | 18 | 4 | 21.6 |
| Myoz1 Myozenin-1 | IPI00459945 | 68 | 644 | 24 | 9 | 50.7 | 74 | 530 | 19 | 9 | 46.5 |
| Hba-a1;Hba-a2 Putative uncharacterized protein | IPI00110658 | 69 | 637 | 24 | 5 | 31.7 | 75 | 502 | 17 | 11 | 11.7 |
| Pgk1 Phosphoglycerate kinase 1 | IPI00555069 | 71 | 613 | 28 | 11 | 37.2 | 77 | 478 | 15 | 10 | 17.8 |
| Got1 Aspartate aminotransferase, cytoplasmic | IPI00230204 | 73 | 597 | 23 | 8 | 30.5 | 78 | 441 | 14 | 9 | 40.7 |
| Got2 Aspartate aminotransferase, mitochondrial | IPI00117312 | 75 | 573 | 20 | 7 | 19.1 | 80 | 438 | 11 | 8 | 16.0 |
| Eef1a2 Elongation factor 1-alpha 2 | IPI00119667 | 76 | 553 | 17 | 7 | 21.4 | 81 | 437 | 14 | 7 | 16.6 |
| Ldb3 LIM domain-binding protein 3 isoform c | IPI00403040 | 77 | 547 | 28 | 10 | 24.3 | 83 | 401 | 15 | 8 | 22.5 |
| Uqcrc2 Cytochrome b-c1 complex subunit 2, mitochondrial | IPI00119138 | 78 | 502 | 23 | 10 | 32.0 | 84 | 375 | 12 | 7 | 30.5 |
| Pgam2 Phosphoglycerate mutase 2 | IPI00230706 | 79 | 489 | 23 | 9 | 37.2 | 85 | 373 | 10 | 6 | 10.0 |
| Ndufs1 NADH-ubiquinone oxidoreductase 75 kDa subunit, mitochondrial | IPI00308882 | 80 | 487 | 21 | 10 | 19.1 | 86 | 365 | 18 | 4 | 23.3 |
| Uqcrc1 Cytochrome b-c1 complex subunit 1, mitochondrial | IPI00111885 | 81 | 483 | 16 | 8 | 25.0 | 87 | 353 | 14 | 8 | 27.6 |
| Myom1 Isoform 2 of Myomesin-1 | IPI00281109 | 83 | 478 | 23 | 14 | 13.5 | 88 | 353 | 8 | 5 | 19.5 |
| Slc25a12 Calcium-binding mitochondrial carrier protein Aralar1 | IPI00308162 | 84 | 473 | 22 | 10 | 22.5 | 89 | 351 | 13 | 6 | 20.7 |
| Gpi1 Glucose-6-phosphate isomerase | IPI00228633 | 86 | 467 | 21 | 12 | 27.2 | 90 | 343 | 10 | 6 | 17.3 |
| Vdac3 Voltage-dependent anion-selective channel protein 3 | IPI00122548 | 89 | 413 | 18 | 4 | 15.5 | 91 | 341 | 12 | 5 | 15.2 |
| Adssl1 Isoform 1 of Adenylosuccinate synthetase isozyme 1 | IPI00123190 | 91 | 399 | 12 | 7 | 21.4 | 92 | 327 | 10 | 3 | 23.9 |
| Hspa8 Heat shock cognate 71 kDa protein | IPI00323357 | 92 | 398 | 18 | 12 | 17.6 | 93 | 326 | 9 | 6 | 15.9 |
| Trf Serotransferrin | IPI00139788 | 93 | 397 | 20 | 14 | 25.8 | 94 | 312 | 7 | 5 | 7.4 |
| Bgn Biglycan | IPI00123194 | 94 | 396 | 19 | 8 | 24.4 | 95 | 310 | 11 | 7 | 18.7 |
| Ldhb L-lactate dehydrogenase B chain | IPI00229510 | 95 | 382 | 7 | 4 | 15.9 | 96 | 274 | 11 | 4 | 19.1 |
| Cs Citrate synthase, mitochondrial | IPI00113141 | 96 | 373 | 15 | 5 | 12.7 | 98 | 262 | 9 | 4 | 3.2 |
| Mdh1 Malate dehydrogenase, cytoplasmic | IPI00336324 | 97 | 361 | 14 | 7 | 24.9 | 99 | 253 | 10 | 3 | 22.1 |
| Idh3a Isoform 1 of Isocitrate dehydrogenase [NAD] subunit alpha, mitochondrial | IPI00459725 | 98 | 358 | 11 | 7 | 24.0 | 101 | 245 | 10 | 3 | 11.8 |
| H2afj Histone H2A.J | IPI00153400 | 101 | 350 | 9 | 3 | 27.1 | 102 | 225 | 8 | 4 | 13.7 |
| mt-Co2 Cytochrome c oxidase subunit 2 | IPI00131176 | 102 | 336 | 11 | 5 | 26.4 | 103 | 220 | 9 | 6 | 19.1 |
| Pfkm 6-phosphofructokinase, muscle type | IPI00331541 | 103 | 327 | 12 | 8 | 13.5 | 104 | 220 | 8 | 5 | 16.2 |
| Aco2 Aconitate hydratase, mitochondrial | IPI00116074 | 104 | 321 | 24 | 11 | 16.8 | 105 | 216 | 9 | 6 | 8.1 |
| Serpina1e Alpha-1-antitrypsin 1-5 | IPI00123927 | 105 | 318 | 10 | 4 | 16.7 | 106 | 212 | 5 | 3 | 12.5 |
| Pgm1;Pgm2 Phosphoglucomutase-1 | IPI00555140 | 106 | 316 | 11 | 9 | 21.9 | 107 | 212 | 5 | 1 | 4.9 |
| Dlat Dihydrolipoyllysine-residue acetyltransferase component of pyruvate dehydrogenase complex, mitochondrial | IPI00153660 | 107 | 309 | 11 | 7 | 15.9 | 108 | 203 | 4 | 2 | 8.3 |
| Anxa5 Annexin A5 | IPI00317309 | 108 | 300 | 8 | 6 | 21.9 | 110 | 198 | 10 | 2 | 9.3 |
| Pdhb Pyruvate dehydrogenase E1 component subunit beta, mitochondrial | IPI00132042 | 109 | 297 | 12 | 5 | 19.8 | 111 | 198 | 3 | 2 | 7.0 |
| Ywhag 14-3-3 protein gamma | IPI00230707 | 110 | 286 | 8 | 4 | 21.5 | 112 | 197 | 11 | 4 | 8.6 |
| Tuba1b Tubulin alpha-1B chain | IPI00117348 | 111 | 269 | 15 | 7 | 22.0 | 113 | 194 | 4 | 4 | 4.0 |
| Car3 Carbonic anhydrase 3 | IPI00221890 | 112 | 269 | 18 | 8 | 36.2 | 114 | 192 | 11 | 8 | 4.2 |
| Tuba4a Tubulin alpha-4A chain | IPI00117350 | 113 | 266 | 14 | 6 | 20.1 | 115 | 190 | 7 | 6 | 19.2 |
| Atp5o;LOC100047429 ATP synthase subunit O, mitochondrial | IPI00118986 | 114 | 265 | 12 | 6 | 43.2 | 117 | 180 | 6 | 4 | 17.7 |
| Pebp1 Phosphatidylethanolamine-binding protein 1 | IPI00137730 | 115 | 258 | 4 | 3 | 28.3 | 118 | 177 | 4 | 3 | 8.7 |
| Aldoc Fructose-bisphosphate aldolase C | IPI00119458 | 118 | 247 | 8 | 5 | 15.7 | 119 | 174 | 5 | 3 | 11.8 |
| Gdi2 Isoform 1 of Rab GDP dissociation inhibitor beta | IPI00122565 | 119 | 228 | 4 | 2 | 7.6 | 120 | 167 | 3 | 2 | 9.3 |
| Uba52 Ubiquitin-60S ribosomal protein L40 | IPI00138892 | 121 | 207 | 5 | 1 | 12.5 | 121 | 165 | 4 | 3 | 6.1 |
| Dlst Isoform 1 of Dihydrolipoyllysine-residue succinyltransferase component of 2-oxoglutarate dehydrogenase complex, mitochondrial | IPI00134809 | 122 | 207 | 5 | 4 | 11.5 | 122 | 165 | 3 | 3 | 15.6 |
| Etfa Electron transfer flavoprotein subunit alpha, mitochondrial | IPI00116753 | 123 | 204 | 8 | 7 | 28.8 | 123 | 162 | 6 | 3 | 16.1 |
| Sdhb Succinate dehydrogenase [ubiquinone] iron-sulfur subunit, mitochondrial | IPI00338536 | 124 | 203 | 2 | 2 | 7.4 | 124 | 161 | 3 | 3 | 9.8 |
| Ndufs3 NADH dehydrogenase [ubiquinone] iron-sulfur protein 3, mitochondrial | IPI00121309 | 126 | 200 | 5 | 3 | 14.1 | 125 | 149 | 6 | 4 | 15.3 |
| Etfb Electron transfer flavoprotein subunit beta | IPI00121440 | 127 | 199 | 7 | 5 | 21.6 | 126 | 142 | 6 | 6 | 6.5 |
| Serpina1c Alpha-1-antitrypsin 1-3 | IPI00123920 | 128 | 198 | 18 | 6 | 26.4 | 127 | 136 | 6 | 4 | 7.7 |
| Pdha1 Pyruvate dehydrogenase E1 component subunit alpha, somatic form, mitochondrial | IPI00337893 | 129 | 195 | 8 | 5 | 16.7 | 128 | 133 | 7 | 5 | 22.9 |
| Ywhae 14-3-3 protein epsilon | IPI00118384 | 130 | 192 | 10 | 5 | 25.9 | 129 | 130 | 2 | 1 | 14.9 |
| Capza2 F-actin-capping protein subunit alpha-2 | IPI00111265 | 131 | 192 | 6 | 4 | 15.7 | 130 | 127 | 6 | 4 | 9.5 |
| Col6a2 Collagen alpha-2(VI) chain | IPI00621027 | 135 | 178 | 9 | 7 | 10.1 | 131 | 123 | 2 | 1 | 3.6 |
| Rtn2 Isoform 1 of Reticulon-2 | IPI00120427 | 137 | 167 | 7 | 3 | 11.7 | 132 | 123 | 5 | 4 | 19.8 |
| Acat1 Acetyl-CoA acetyltransferase, mitochondrial | IPI00154054 | 138 | 166 | 5 | 3 | 12.7 | 133 | 122 | 8 | 2 | 21.7 |
| Ugp2 Isoform 1 of UTP--glucose-1-phosphate uridylyltransferase | IPI00131204 | 139 | 163 | 6 | 6 | 15.7 | 134 | 121 | 3 | 3 | 8.4 |
| Hist1h4m;Hist1h4c;Hist1h4k;Hist1h4h;Hist2h4;Hist1h4b;Hist1h4j;Hist1h4i;Hist1h4d;Hist1h4a;Hist1h4f;Hist4h4;Hist1h4n Histone H4 | IPI00407339 | 140 | 156 | 10 | 2 | 17.5 | 136 | 117 | 7 | 3 | 7.7 |
| Ndrg2 Isoform 1 of Protein NDRG2 | IPI00136134 | 142 | 155 | 4 | 3 | 16.4 | 137 | 116 | 3 | 3 | 17.6 |
| Sucla2 Succinyl-CoA ligase [ADP-forming] subunit beta, mitochondrial | IPI00261627 | 146 | 151 | 7 | 6 | 16.0 | 139 | 110 | 5 | 5 | 13.8 |
| Prdx2 Peroxiredoxin-2 | IPI00117910 | 149 | 145 | 2 | 2 | 16.7 | 140 | 107 | 3 | 3 | 10.8 |
| Tufm Isoform 1 of Elongation factor Tu, mitochondrial | IPI00274407 | 150 | 145 | 2 | 2 | 6.6 | 142 | 105 | 3 | 1 | 6.5 |
| Anxa2 Annexin A2 | IPI00468203 | 151 | 139 | 10 | 7 | 22.7 | 143 | 103 | 3 | 2 | 10.2 |
| Flnc Uncharacterized protein | IPI00664670 | 152 | 138 | 4 | 4 | 3.0 | 145 | 102 | 2 | 2 | 19.1 |
| Hspb6 Heat shock protein beta-6 | IPI00128791 | 153 | 138 | 4 | 4 | 38.3 | 146 | 102 | 3 | 2 | 6.9 |
| Atp5k ATP synthase subunit e, mitochondrial | IPI00111770 | 154 | 137 | 2 | 2 | 32.4 | 148 | 100 | 4 | 4 | 10.2 |
| Tmem38a Trimeric intracellular cation channel type A | IPI00128227 | 155 | 137 | 2 | 1 | 6.0 | 149 | 97 | 2 | 1 | 8.4 |
| Gpd1 Glycerol-3-phosphate dehydrogenase [NAD+], cytoplasmic | IPI00230185 | 156 | 133 | 4 | 4 | 14.9 | 150 | 95 | 3 | 2 | 16.4 |
| Kbtbd10 Uncharacterized protein | IPI00136119 | 158 | 127 | 7 | 7 | 13.7 | 151 | 94 | 3 | 3 | 4.8 |
| Slc25a11 Mitochondrial 2-oxoglutarate/malate carrier protein | IPI00230754 | 160 | 123 | 5 | 4 | 19.7 | 152 | 93 | 2 | 2 | 14.1 |
| Vdac2 Voltage-dependent anion-selective channel protein 2 | IPI00122547 | 161 | 116 | 8 | 6 | 24.1 | 154 | 91 | 1 | 1 | 3.5 |
| Ptrf Polymerase I and transcript release factor | IPI00117689 | 162 | 116 | 2 | 1 | 4.6 | 155 | 91 | 1 | 1 | 3.2 |
| Dld Dihydrolipoyl dehydrogenase | IPI00331564 | 163 | 116 | 5 | 5 | 12.5 | 156 | 90 | 3 | 3 | 32.3 |
| Akr1b10 aldo-keto reductase family 1, member B10 | IPI00222284 | 169 | 108 | 1 | 1 | 3.5 | 159 | 84 | 2 | 1 | 5.2 |
| Pdlim5 Isoform 2 of PDZ and LIM domain protein 5 | IPI00415684 | 173 | 108 | 1 | 1 | 4.7 | 160 | 82 | 2 | 1 | 4.9 |
| Gstp1 Glutathione S-transferase P 1 | IPI00555023 | 175 | 102 | 3 | 2 | 12.9 | 161 | 80 | 1 | 1 | 16.9 |
| Hspd1 Isoform 1 of 60 kDa heat shock protein, mitochondrial | IPI00308885 | 177 | 99 | 5 | 5 | 14.1 | 163 | 80 | 1 | 1 | 5.4 |
| Akr1b3 Aldose reductase | IPI00223757 | 179 | 97 | 7 | 3 | 10.8 | 164 | 80 | 1 | 1 | 4.6 |
| Ogn Mimecan | IPI00120848 | 180 | 96 | 9 | 5 | 18.8 | 165 | 80 | 1 | 1 | 3.6 |
| Sh3bgr SH3 domain-binding glutamic acid-rich protein | IPI00125512 | 181 | 95 | 3 | 2 | 14.5 | 166 | 80 | 1 | 1 | 5.4 |
| Cfl2 Cofilin-2 | IPI00266188 | 182 | 94 | 5 | 4 | 30.7 | 168 | 80 | 1 | 1 | 14.8 |
| Cryab Alpha-crystallin B chain | IPI00138274 | 183 | 93 | 4 | 3 | 28.6 | 169 | 80 | 1 | 1 | 2.0 |
| Atp5c1 ATP synthase subunit gamma, mitochondrial | IPI00313475 | 186 | 82 | 5 | 3 | 10.4 | 170 | 80 | 4 | 2 | 9.8 |
| Ndufa9 NADH dehydrogenase [ubiquinone] 1 alpha subcomplex subunit 9, mitochondrial | IPI00120212 | 187 | 80 | 5 | 4 | 19.4 | 171 | 76 | 6 | 4 | 10.4 |
| Slc25a3 Phosphate carrier protein, mitochondrial | IPI00124771 | 188 | 78 | 5 | 3 | 8.4 | 172 | 76 | 3 | 1 | 12.5 |
| Neb Uncharacterized protein | IPI00720238 | 190 | 76 | 8 | 8 | 1.7 | 173 | 73 | 6 | 4 | 17.1 |
| Prdx3 Thioredoxin-dependent peroxide reductase, mitochondrial | IPI00116192 | 192 | 73 | 3 | 3 | 14.4 | 174 | 71 | 1 | 1 | 4.1 |
| Atp5j2 ATP synthase subunit f, mitochondrial | IPI00271986 | 193 | 66 | 4 | 1 | 13.6 | 175 | 68 | 5 | 2 | 17.5 |
| Mtch2 Mitochondrial carrier homolog 2 | IPI00132039 | 194 | 65 | 2 | 1 | 7.9 | 176 | 64 | 6 | 6 | 9.7 |
| Hspb1 Isoform A of Heat shock protein beta-1 | IPI00128522 | 195 | 65 | 4 | 3 | 27.8 | 177 | 64 | 5 | 5 | 10.3 |
| Acadvl Very long-chain specific acyl-CoA dehydrogenase, mitochondrial | IPI00119203 | 196 | 64 | 3 | 3 | 8.2 | 178 | 61 | 1 | 1 | 5.8 |
| Hadhb Trifunctional enzyme subunit beta, mitochondrial | IPI00115607 | 197 | 64 | 4 | 3 | 6.5 | 179 | 59 | 1 | 1 | 5.2 |
| Cox5a Cytochrome c oxidase subunit 5A, mitochondrial | IPI00120719 | 199 | 63 | 3 | 2 | 16.4 | 181 | 58 | 7 | 1 | 0.3 |
| Oxct1 Succinyl-CoA:3-ketoacid-coenzyme A transferase 1, mitochondrial | IPI00132653 | 201 | 62 | 3 | 3 | 11.3 | 182 | 54 | 2 | 1 | 8.0 |
| Padi2 Protein-arginine deiminase type-2 | IPI00123014 | 202 | 62 | 2 | 2 | 3.4 | 183 | 53 | 10 | 1 | 1.1 |
| Atp5d ATP synthase subunit delta, mitochondrial | IPI00453777 | 203 | 62 | 1 | 1 | 8.3 | 184 | 51 | 3 | 2 | 12.9 |
| Pde4d Uncharacterized protein | IPI00458190 | 210 | 56 | 13 | 1 | 1.1 | 185 | 50 | 4 | 4 | 7.5 |
| Fh1 Isoform Mitochondrial of Fumarate hydratase, mitochondrial | IPI00129928 | 213 | 54 | 2 | 2 | 4.9 | 186 | 50 | 2 | 1 | 6.3 |
| Apobec2 Probable C->U-editing enzyme APOBEC-2 | IPI00125150 | 214 | 54 | 3 | 2 | 9.8 | 187 | 48 | 1 | 1 | 2.8 |
| Ndufa12 NADH dehydrogenase (Ubiquinone) 1 alpha subcomplex, 12 | IPI00344004 | 217 | 51 | 2 | 2 | 19.5 | 188 | 48 | 2 | 1 | 5.6 |
| Hadha Trifunctional enzyme subunit alpha, mitochondrial | IPI00223092 | 219 | 51 | 2 | 1 | 2.8 | 189 | 47 | 1 | 1 | 6.6 |
| Hhatl Protein-cysteine N-palmitoyltransferase HHAT-like protein | IPI00315964 | 220 | 51 | 2 | 2 | 4.4 | 190 | 47 | 3 | 2 | 19.3 |
| Hsp90ab1 Heat shock protein HSP 90-beta | IPI00554929 | 221 | 50 | 3 | 3 | 5.0 | 192 | 43 | 3 | 2 | 9.7 |
| Myot Myotilin | IPI00120508 | 222 | 50 | 4 | 4 | 9.1 | 193 | 41 | 4 | 3 | 11.1 |
| Tomm22 Mitochondrial import receptor subunit TOM22 homolog | IPI00315135 | 230 | 49 | 1 | 1 | 14.8 | 194 | 39 | 2 | 2 | 8.0 |
| Sod2 Superoxide dismutase [Mn], mitochondrial | IPI00109109 | 231 | 49 | 2 | 1 | 6.3 | 195 | 38 | 2 | 2 | 1.6 |
| Sypl2 Putative uncharacterized protein | IPI00131500 | 233 | 48 | 3 | 2 | 5.6 | 196 | 38 | 1 | 1 | 3.0 |
| Rpsa;LOC100505031 40S ribosomal protein SA | IPI00123604 | 234 | 47 | 2 | 1 | 5.8 | 197 | 35 | 3 | 3 | 8.9 |
| Eef2 Elongation factor 2 | IPI00466069 | 235 | 47 | 3 | 3 | 3.6 | 198 | 34 | 1 | 1 | 5.9 |
| Dci enoyl-CoA delta isomerase 1, mitochondrial precursor | IPI00331692 | 238 | 42 | 2 | 1 | 5.2 | 199 | 34 | 1 | 1 | 1.9 |
| Acaa2 3-ketoacyl-CoA thiolase, mitochondrial | IPI00226430 | 239 | 40 | 3 | 3 | 9.6 | 200 | 33 | 1 | 1 | 10.9 |
| Sdha Succinate dehydrogenase [ubiquinone] flavoprotein subunit, mitochondrial | IPI00230351 | 240 | 39 | 3 | 3 | 5.7 | 201 | 31 | 4 | 3 | 16.5 |
| Suclg1 Succinyl-CoA ligase [GDP-forming] subunit alpha, mitochondrial | IPI00406442 | 241 | 38 | 3 | 1 | 4.9 | 202 | 30 | 2 | 2 | 10.6 |
| Atp5h ATP synthase subunit d, mitochondrial | IPI00230507 | 243 | 37 | 3 | 3 | 29.2 | 203 | 30 | 2 | 2 | 32.7 |
| 9530053A07Rik Uncharacterized protein | IPI00227522 | 244 | 36 | 5 | 1 | 0.3 | 204 | 29 | 2 | 1 | 4.3 |
| Trim72 Tripartite motif-containing protein 72 | IPI00462157 | 246 | 36 | 2 | 2 | 5.9 | 205 | 29 | 2 | 1 | 3.6 |
| 2210010C04Rik trypsinogen 7 | IPI00131674 | 248 | 35 | 4 | 2 | 8.1 | 207 | 27 | 1 | 1 | 1.8 |
| Il5ra Interleukin-5 receptor subunit alpha | IPI00120194 | 249 | 33 | 12 | 1 | 3.1 | 208 | 27 | 1 | 1 | 7.0 |
| Lama2 laminin subunit alpha-2 | IPI00756745 | 257 | 33 | 1 | 1 | 0.5 | 209 | 27 | 1 | 1 | 5.0 |
| Ndufv2 Isoform 1 of NADH dehydrogenase [ubiquinone] flavoprotein 2, mitochondrial | IPI00169925 | 258 | 33 | 3 | 2 | 8.5 | 210 | 26 | 1 | 1 | 8.1 |
| Phb2 Prohibitin-2 | IPI00321718 | 261 | 31 | 2 | 2 | 7.4 | 211 | 25 | 4 | 1 | 3.1 |
| Ndufb10 NADH dehydrogenase [ubiquinone] 1 beta subcomplex subunit 10 | IPI00121288 | 267 | 27 | 2 | 2 | 18.8 | 212 | 25 | 2 | 2 | 5.6 |
| Nme2 Nucleoside diphosphate kinase B | IPI00127417 | 270 | 26 | 1 | 1 | 7.9 | 214 | 24 | 1 | 1 | 2.6 |
| Hist2h3b;Hist1h3e;Hist1h3c;Hist2h3c2-ps;Hist1h3f;Hist2h3c1;Hist1h3b;Hist1h3d Histone H3.2 | IPI00230730 | 272 | 26 | 2 | 1 | 5.1 | 217 | 24 | 1 | 1 | 4.3 |
| Atp1b1 Sodium/potassium-transporting ATPase subunit beta-1 | IPI00121550 | 273 | 26 | 1 | 1 | 3.6 | 220 | 24 | 1 | 1 | 8.3 |
| Atad3a Isoform 1 of ATPase family AAA domain-containing protein 3 | IPI00126913 | 274 | 25 | 5 | 1 | 1.7 | 221 | 24 | 2 | 2 | 3.4 |
| Tcap Telethonin | IPI00119331 | 279 | 24 | 1 | 1 | 6.6 | 222 | 23 | 2 | 1 | 14.7 |
| Ndufv1 NADH dehydrogenase [ubiquinone] flavoprotein 1, mitochondrial | IPI00130460 | 280 | 24 | 1 | 1 | 1.9 | 224 | 23 | 3 | 1 | 1.7 |
| Vcp Transitional endoplasmic reticulum ATPase | IPI00622235 | 292 | 23 | 2 | 2 | 3.1 | 225 | 22 | 1 | 1 | 2.1 |
| Decr1 2,4-dienoyl-CoA reductase, mitochondrial | IPI00387379 | 295 | 22 | 1 | 1 | 3.0 | 227 | 21 | 1 | 1 | 13.6 |
| Acsl1 Long-chain-fatty-acid--CoA ligase 1 | IPI00112549 | 299 | 19 | 2 | 2 | 4.3 | 229 | 20 | 1 | 1 | 6.6 |
| Atp5f1 ATP synthase subunit b, mitochondrial | IPI00341282 | 303 | 18 | 2 | 2 | 6.2 | 231 | 19 | 1 | 1 | 3.5 |
| Myoz2 Myozenin-2 | IPI00122334 | 304 | 18 | 1 | 1 | 4.2 | 232 | 19 | 1 | 1 | 0.7 |
| Uqcr10 Cytochrome b-c1 complex subunit 9 | IPI00153381 | 310 | 18 | 1 | 1 | 10.9 | 233 | 19 | 1 | 1 | 0.5 |
| Fhl1 Isoform 1 of Four and a half LIM domains protein 1 | IPI00309997 | 313 | 18 | 1 | 1 | 4.3 | 234 | 19 | 2 | 1 | 0.8 |
| Myom3 Myomesin-3 | IPI00378974 | 315 | 18 | 1 | 1 | 1.2 | 235 | 18 | 1 | 1 | 5.8 |
| Vcl Vinculin | IPI00405227 | 316 | 18 | 1 | 1 | 1.0 | 237 | 17 | 1 | 1 | 17.2 |
| Alox15 Arachidonate 12-lipoxygenase, leukocyte-type | IPI00553304 | 318 | 18 | 1 | 1 | 3.0 | 238 | 17 | 1 | 1 | 1.1 |
| Dsp Desmoplakin | IPI00553419 | 324 | 16 | 1 | 1 | 0.2 | 239 | 17 | 1 | 1 | 3.7 |
| Agl Uncharacterized protein | IPI00662244 | 326 | 16 | 3 | 3 | 2.7 | 242 | 17 | 1 | 1 | 8.1 |
| Tmod4 Tropomodulin-4 | IPI00124328 | 327 | 16 | 2 | 2 | 5.8 | 243 | 17 | 1 | 1 | 3.0 |
| Asap2 Isoform 1 of Arf-GAP with SH3 domain, ANK repeat and PH domain-containing protein 2 | IPI00355808 | 328 | 16 | 2 | 1 | 0.8 | 245 | 17 | 1 | 1 | 5.1 |
| Atp5l;Gm10221 Putative uncharacterized protein | IPI00133342 | 329 | 16 | 2 | 1 | 14.7 | 247 | 17 | 1 | 1 | 1.6 |
| Dnajc24 Uncharacterized protein | IPI00751149 | 330 | 16 | 2 | 1 | 17.2 | 248 | 17 | 1 | 1 | 1.6 |
| Bcl11a Isoform 8 of B-cell lymphoma/leukemia 11A | IPI00267748 | 345 | 14 | 1 | 1 | 3.1 | 251 | 17 | 1 | 1 | 2.7 |
| Trmt5 Isoform 1 of tRNA (guanine-N(1)-)-methyltransferase | IPI00331418 | 349 | 14 | 1 | 1 | 1.6 | 252 | 17 | 1 | 1 | 1.2 |
| Gcfc1 Uncharacterized protein | IPI01008165 | 354 | 14 | 1 | 1 | 6.9 | 253 | 17 | 1 | 1 | 23.5 |
| Bsg Isoform 2 of Basigin | IPI00113869 | 357 | 14 | 1 | 1 | 3.7 | 254 | 17 | 1 | 1 | 6.9 |
| Ndufa10 NADH dehydrogenase [ubiquinone] 1 alpha subcomplex subunit 10, mitochondrial | IPI00116748 | 358 | 14 | 1 | 1 | 3.1 | 257 | 17 | 1 | 1 | 4.1 |
| Acadl Long-chain specific acyl-CoA dehydrogenase, mitochondrial | IPI00119114 | 359 | 14 | 1 | 1 | 2.8 | 259 | 17 | 1 | 1 | 3.1 |
| Gm20094 protein transport protein Sec61 subunit gamma-like | IPI00985899 | 364 | 14 | 1 | 1 | 23.5 | 260 | 17 | 1 | 1 | 1.6 |
| Hadh Hydroxyacyl-coenzyme A dehydrogenase, mitochondrial | IPI00121105 | 367 | 14 | 1 | 1 | 3.5 | 264 | 14 | 1 | 1 | 3.0 |
| Ampd1 AMP deaminase 1 | IPI00625079 | 378 | 14 | 1 | 1 | 2.3 | 265 | 14 | 1 | 1 | 4.0 |
| Idh2 Isocitrate dehydrogenase [NADP], mitochondrial | IPI00318614 | 379 | 13 | 1 | 1 | 3.5 | 266 | 13 | 1 | 1 | 4.6 |
