# Supplementary figures and images for: Proteomic analysis of laser capture microscopy purified myotendinous junction regions from muscle sections
Source: Proteome Sci. 2014 May 7;12:25. doi: 10.1186/1477-5956-12-25 (PMC4113200; doi:10.1186/1477-5956-12-25)

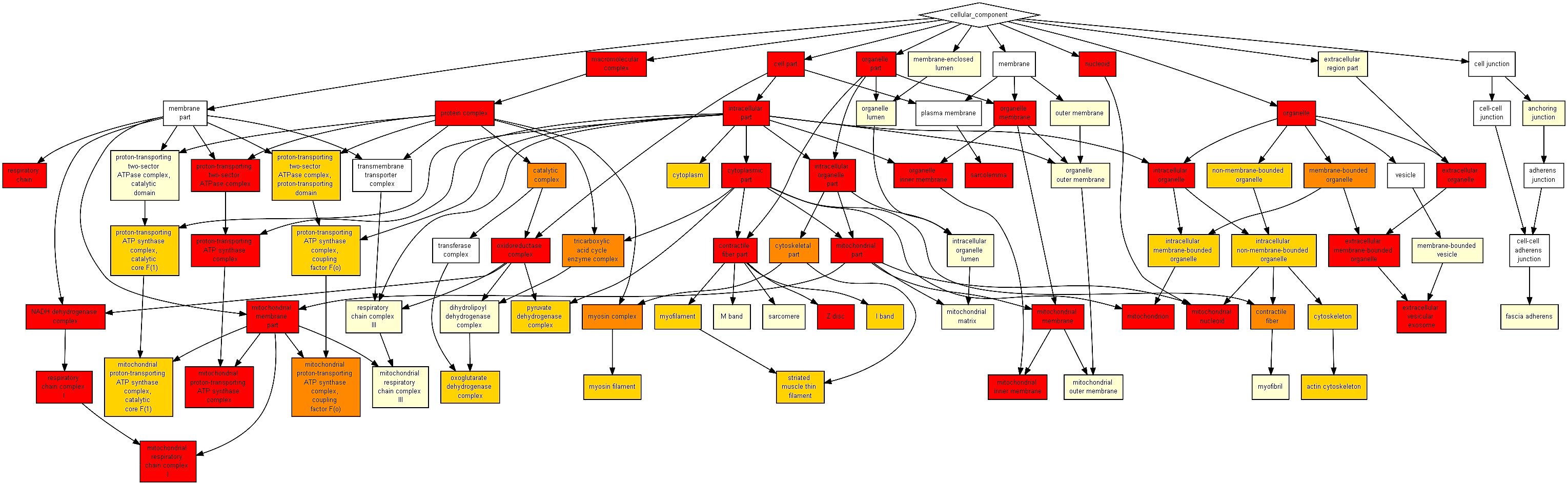

Supplement: Additional file 2: Figure S1 — Blind analysis of the most likely cellular components associated with the proteins identified by LC-MS/MS in the myotendinous junction. A diagram generated by the GOrilla software (see text for details) using all proteins identified in the myotendinous junction sample. The color of the box indicates P value interval of the enclosed term: white, yellow, orange, brown and red denotes P values > 10−3, 10−3 to 10−5, 10−5 to 10−7,10−7 to 10−9 and < 10−9 respectively. The P value is the enrichment p-value computed according to a minimum hypergeometric model and is not corrected for multiple testing (see Additional file 3: Table S2 for corrected P values). [file 1477-5956-12-25-S2.png]
